# Aggregate Consumer Exposure and Risk Assessment in the EU—A Case Study

**DOI:** 10.3390/toxics14020165

**Published:** 2026-02-11

**Authors:** Jan Oltmanns, Christoph Scheibelein, Fabian A. Grimm

**Affiliations:** 1 Forschungs- und Beratungsinstitut Gefahrstoffe GmbH (FoBiG), Klarastraße 63, 79106 Freiburg, Germany; jan.oltmanns@fobig.de; 2 Clariant Plastics & Coatings (Deutschland) GmbH, Ludwig-Hermann-Str. 100, 86368 Gersthofen, Germany; christoph.scheibelein@clariant.com; 3 Clariant Produkte (Deutschland) GmbH, Brüningstr. 50, 65929 Frankfurt am Main, Germany

**Keywords:** consumer exposure, aggregate exposure assessment, risk assessment, European Union, case study

## Abstract

Consumer exposure to chemicals in the EU is currently assessed separately for different products without aggregating exposure from different sources. A more integrated ap proach represents a promising opportunity to improve comprehensive risk evaluation and transparency across the value chain. This study develops aggregate consumer expo sure and risk assessment methods that involve calculation of exposure and risk for each pathway using the risk characterization ratio (RCR) as a uniform risk metric. Aggregate risk is obtained by adding up pathway-specific RCRs. The developed methodology re presents a new approach by evaluating exposure of seven population groups via all path ways and by using key input values normalized to body weight to reflect population-specific differences. The study demonstrates the practical applicability of the methodol o gy by assessing consumer exposure to the antioxidant ethylene bis[3,3-bis(3-tert-butyl-4-hydroxyphenyl)butyrate] (Hostanox^®^ O 3), resulting from its use in food and drinking water contact materials, textiles and sealants. This case study demonstrates aggregate RCRs well below one for all groups. The highest aggregate RCRs are found for infants and toddlers, reflecting their proportionally higher food consumption and skin surface area. The methodology is transparent and can easily be applied to other substances, e.g., by industry stakeholders and authorities, if the substance concentration in products can be established. This study may inform further development of aggregate exposure and risk methods in EU regulatory frameworks.

## 1. Introduction

Transparency across the chemicals value chain represents a fundamental principle for ensuring consumer safety in today’s complex regulatory landscape. Consumers are exposed to chemicals from a variety of sources, and contributing consumer uses are fre quently regulated under different European legislation, which are within the remit of ac tivity of different European agencies. For example, exposure to a substance used in plastic food contact materials (FCMs) is covered by Regulation (EU) No 10/2011 within the remit of the European Food Safety Authority (EFSA), while exposure to the same substance in uses covered by the REACH Regulation (Regulation (EC) No 1907/2006) is within the remit of the European Chemicals Agency (ECHA).

Regulatory area-specific exposure and risk assessment approaches are meaningful in representing situations specific to a certain area but lack assessment opportunities to demonstrate exposure and risk across all relevant regulatory areas.

While the safe use of a chemical may be demonstrated within each domain, aggregate exposure may be associated with a risk to consumers. Such situations go unnoticed because there is no uniform system or platform and, indeed, no harmonized methodology to carry out aggregate exposure and risk assessments in the EU. In contrast, an aggregate risk assessment can provide an important line of evidence in support of science-based risk management decisions.

The Chemicals Strategy for Sustainability (CSS) published by the European Commission in 2020 [1] places an emphasis on the ‘one substance, one assessment’ (OSOA) principle that ‘aims to ensure that methodologies are made more coherent and to the extent possible harmonised.’ One of the tools to achieve this aim is the ‘common data platform on chemicals’ for which legislation has recently been proposed as part of the so-called OSOA package ‘to ensure that the data contained in it are findable, accessible, interoperable and reusable’ [2]. The envisaged data platform, however, places a strong emphasis on data and information related to the hazards of a substance, while the exposure of consumers appears to be primarily addressed by generating biomonitoring data (also see discussion). Importantly, it does not include provisions on how to address aggregate consumer exposure to chemicals resulting from uses covered by different European legislations.

While the legislative proposal is limited to certain aspects, it is evident that chemical risk assessment under the OSOA principle needs to be further developed and ‘has to span across multiple legislative domains and be able to account for different routes and sources of exposure’ [3]. A recent report for EFSA has outlined a roadmap for action to advance aggregate consumer exposure and risk assessments in the EU [4] and has also summarized many of the challenges involved in such a task. In addition to the more general issues identified by these authors, challenges arise from the fact that different population groups are assessed in different regulatory areas with sometimes different anthropometric input data being used in the models and methods to estimate exposure, as discussed in this study by some illustrative examples.

This study provides a quantitative aggregate exposure and risk assessment for consumer exposure to Ethylene bis[3,3-bis(3-tert-butyl-4-hydroxyphenyl)-butyrate] (EC no.: 251-073-2, CAS no.: 32509-66-3; hereinafter “Hostanox^®^ O 3”), an additive used in several applications covered by different EU legislation. In assessing consumer exposure to Hostanox^®^ O 3, this study applies a structured methodology with a detailed and transparent justification of input values used in the exposure and risk assessment. The assessment covers seven clearly defined population groups and provides—to the extent possible—anthropometric input data specifically for these population groups. Importantly, it uses input values normalized to body weight for parameters that rapidly change with age, such as food consumption and the skin surface area. The assessment addresses different pathways and routes of exposure separately and finally provides an aggregated exposure and risk assessment covering all pathways of exposure.

This study intends to contribute to the development of aggregate exposure and risk assessment in the EU by providing a possible methodology and discussing critical aspects. The novelty of this methodology consists of (a) aggregating exposure from different sources currently evaluated separately; (b) covering seven population groups, reflecting the higher exposure potential of children due to anthropometric differences; and (c) using key input values normalized to body weight for most exposure pathways, therefore preventing unrealistic combinations of values for dependent parameters. It demonstrates the general feasibility of an aggregate exposure and risk assessment by providing a substance-specific aggregate exposure and risk assessment for Hostanox^®^ O 3, covering uses currently assessed by different regulatory frameworks under the remit of different regulatory agencies. The case study demonstrates the safe use of the substance even after aggregating exposure from all known consumer uses. The results from the case study are discussed in the context of the generic approach to risk management, the extension of which is also envisaged in the CSS.

## 2. Materials and Methods

### 2.1. Substance Identity, Physico-Chemical Properties and Toxicological Reference Values

Hostanox^®^ O 3 is a solid substance with very low vapor pressure and low water solubility at room temperature. Table 1 summarizes the physicochemical properties from the REACH registration dossier of the lead registrant published in the ECHA CHEM database [5] that are relevant for the exposure assessment. The table also provides the Derived No-Effect Levels (DNELs) for the general population from this dossier that are used in the risk assessment.

Since only upper and lower bound values could be determined for two physico-chemical parameters, the exposure assessment applies a vapor pressure of 1 × 10^−6^ Pa and a log K_ow_ of 7.

The DNELs for the general population were derived from a developmental toxicity study (OECD TG 414) performed according to Good Laboratory Practice (GLP) by the lead registrant according to the provisions in the relevant ECHA Guidance [6]. While a detailed discussion of these reference values is outside the scope of this study, it must be noted that a large assessment factor of 600 was applied in deriving the oral and dermal DNELs, including a factor of 10 for intraspecies differences to cover population groups that are potentially more susceptible than adults (e.g., children and the elderly). Since no specific toxicokinetic information is available, dermal absorption was assumed to be complete (i.e., 100%). While a lower dermal absorption may be justified based on the relevant ECHA Guidance [7] due to the molecular weight above 500 g/mol in combination with a log Kow above 4, these properties may also limit oral absorption. Overall, complete absorption via all pathways was therefore assumed.

EFSA provides a Tolerable Daily Intake (TDI) of 0.1 mg/(kg bw × d) [8] that is based on an identical TDI reported in 1998 by the then Scientific Committee on Food (SCF) of the European Commission [9]. While this TDI is somewhat lower than the oral DNEL, the cited documents do not provide any information on its derivation (e.g., source study, test method, and purity of the test material). The risk assessment is therefore based on the DNELs provided in Table 1.

The TDI mentioned above was used to derive a Specific Migration Limit (SML) of 6 mg/kg food for plastic FCM assuming that 1 kg of food is consumed per day by an adult with a body weight of 60 kg [10]. This SML is included in the relevant legislation (Regu la tion (EU) No 10/2011). From this SML, a Maximum Tolerable Concentration at the tap (MTCtap) of 0.3 mg/L was derived for Hostanox^®^ O 3 that is included in the Commission Implementing Decision (EU) 2024/367, i.e., the EU positive list (EUPL) of substances for drinking water contact material (see Supplementary Materials File (SMF), Section S1.1, for details). The MTCtap is used in the exposure assessment as described below.

It is noted that ECHA’s Committee for Risk Assessment (RAC) recently recom men ded a harmonized classification of the substance due to developmental toxicity as a repro duc tive toxicant in Category 1B according to the European CLP Regulation (Regulation (EC) No 1272/2008). The European Commission has not yet decided on this case. Since this study is not concerned with the hazard assessment of the substance and the respective classification proposal, the information underlying the proposed classification is not discussed further. It is, however, noted that the derived DNELs cover the endpoint of developmental toxicity.

### 2.2. Identification of Consumer Uses

Hostanox^®^ O 3 functions as a phenolic antioxidant primarily employed for polymer protection against various stressors. The compound demonstrates enhanced resistance to hydrolysis and provides effective long-term heat stabilization in multiple polymer systems, including polyolefins, polyamides, and silyl modified polymers, as well as in resins and articles manufactured from these materials. Notably, Hostanox^®^ O 3 exhibits suitability for applications where the final product may encounter extractive media, specifically water, chlorinated media, fat, and sweat.

The substance is primarily added to products used in industrial and professional settings. Consumer products containing Hostanox^®^ O 3 were identified from publicly available sources (e.g., based on the FCM legislation discussed above) and in-house market sector knowledge. The following applications and products were identified with respect to potential consumer exposure:

- Use in chemical mixtures applied as in-can coating for food (i.e., FCM)
- Use as an additive for elastane fibres that are used in the manufacture of textiles (e.g., bathing suits, leggings, underwear and nappies)
- Use as a chlorine resistant antioxidant in plastic water pipes and tanks
- Use in mixtures that provide heat and light stabilization to sealants, which may be available to consumers as Do-It-Yourself (DIY) products.

Note in this context that terms such as ‘consumers’, ‘consumer uses’ and ‘consumer product’ are used differently in different regulatory areas. We use these terms in a wider sense and refer here to consumer uses, although consumers generally do not ‘use’ materials that come in contact with drinking water, such as pipes.

Although not identified as uses, consumer exposure to two additional products was modelled to address the potential uncertainty in the identification for consumer uses. This uncertainty stems from the fact that the manufacturer of the substance may not be aware of all possible consumer uses due to limited reporting up the supply chain (i.e., from product manufacturers to substance manufacturers).

### 2.3. Population Groups Covered by the Assessment

This exposure and risk assessment covers seven population groups of different ages as defined by EFSA [11]. This approach was chosen for several reasons. First, exposure assessments as performed under the REACH Regulation [12] or for textiles [13] primarily address exposure of adults with only occasional consideration of children. The approach applied here addresses exposure of children as well as the (very) elderly in a systematic way to cover potentially more vulnerable populations. Second, the group of ‘children’—if assessed at all—is often covered by a single group of ‘children’ with different anthropometric characteristics in exposure assessment tools. For example, the lower tier tool frequently used for exposure assessments under the REACH Regulation (the Targeted Risk Assessment (TRA) tool [14,15,16] developed by the European Centre for Toxicology and Ecotoxicology of Chemicals (ECETOC)) assumes a body weight for children of 10.6 kg (no age given), while the higher tier tool ConsExpo (developed by the Dutch National Institute for Public Health and the Environment (Rijksinstituut voor Volksgezondheid en Milieu, RIVM) assumes a body weight of 12.4 kg for children 2–3 years of age [17]. Our approach involving three groups of children covers the change in anthropometric characteristics (e.g., decrease of the skin surface to body weight ratio with age) and food consumption due to rapid growth. At the same time, it ensures that consistent input values are used for each population group. Third, food and drinking water consumption data are available for these seven population groups, making such an approach feasible in the first place. Finally, using these defined population groups allows us to apply the same approach across regulatory boundaries, which is a necessary requirement in an aggregate exposure assessment. As will be discussed below, data are not available for all input parameters for these seven population groups, but this limitation can be adequately accounted for. Overall, the approach applied here ensures consistency with respect to the population groups covered and the anthropometric input values used in the exposure and risk assessment. Table 2 summarizes the ages of the seven population groups as defined in EFSA [11].

Body weights are not provided for these seven population groups, since key input values normalized to body weight are used for all exposure pathways except for the consumer use of sealants.

### 2.4. Input Data for Consumer Exposure Information

#### 2.4.1. Food and Drinking Water Contact Materials

##### Canned Food and Drinking Water: Hostanox^®^ O 3 Concentration

Chronic oral consumer exposure is assessed by combining assumptions on the con centration of Hostanox^®^ O 3 in canned food and drinking water with consumption data.

The concentration of Hostanox^®^ O 3 in canned food items assumed in the exposure assessment is based on unpublished migration studies performed in 2005 in the context of the European FCM authorization [18]. Based on these studies, mean and maximum concentrations in canned food of 0.2 and 0.5 mg/kg, respectively, are assumed in the exposure assessment. The mean concentration reflects the mean migration observed in the full-scale migration study for the only simulant for which migration was detected. The maximum concentration reflects the highest limit of detection in migration studies (see SMF, Section S1.2.1). While the maximum concentration is about an order of magnitude lower than the SML of 6 mg/kg listed in Commission Regulation (EU) No 10/2011, this value is identical to the water solubility of Hostanox^®^ O 3 (Table 1). Since consumption of soft drinks is higher than the consumption of other canned food for most population groups (SMF, Table S1), and because canned food items may also involve aqueous solutions, a maximum Hostanox^®^ concentration equalling its water solubility is considered a meaningful upper end estimate.

The concentration in drinking water is based on the MTCtap of 0.3 mg/L in the EUPL (see above). This value is used as a maximum concentration in the exposure assessment, and one half of this value (0.15 mg/L) is taken as the mean concentration. The MTCtap is just slightly below the water solubility of Hostanox^®^ O 3 of 0.5 mg/L (Table 1).

##### Canned Food and Drinking Water: Consumption Data

Consumption data were extracted from the EFSA Comprehensive European Food Consumption Database [19], referred to below as ‘Comprehensive Database’. The use of the Comprehensive Database was also recently recommended by EFSA and the European Medicines Agency (EMA) with a view of harmonizing dietary exposure assessments across regulatory areas in the EU [20].

The methodology for data extraction and evaluation is complex. It is therefore described in detail in the SMF (Section S1.2.2). Briefly, consumption data were extracted for each population group (Table 2) and each EU Member State (MS) with available data. The extraction covered (a) canned food items, (b) soft drinks and (c) drinking water. The separate extraction and evaluation of canned food items and soft drinks is based on the structure of the Comprehensive Database that reports consumption data aggregated at seven different ‘exposure hierarchy levels’ (L1–L7) in EFSA’s terminology. L1 represents the most aggregated level (e.g., ‘fruit and fruit products’), and L7 represents the most detailed level (e.g., ‘canned or jarred pineapple’). Consumption of canned food is estimated based on relevant L4 entries (e.g., ‘canned or jarred fruit’). As shown by this example, some canned food items indeed represent food in cans or jars, and assuming that all food consumed is in cans, is therefore conservative. Consumption of relevant L4 entries was added up per MS and population group.

For soft drinks, the Comprehensive Database includes a single L3 entry (‘soft drinks’) for which consumption data were extracted. It must be noted that a differentiation of canned soft drinks vs. other soft drinks is impossible. Finally, two L3 entries (‘drinking water’ and ‘unbottled water’) are potentially relevant, and consumption data were extracted for the entry showing the largest fraction of consumers (i.e., fraction consuming the item among all participants in the survey).

Apart from the specific food and drinking water items covered, data extraction followed some general principles that are discussed in more detail in Section S1.2.2 of the SMF. Briefly,

- Only data from the most recent survey were extracted for each MS and population group;
- Consumption data were extracted for consumers only rather than all subjects in the survey;
- Consumption data normalized to body weight (in g food/(kg bw × d) rather than per capita (in g food/d) were extracted;
- Only population groups that have data from more than five MSs were included in the extraction to increase the representativeness of the assessment.

The extracted consumption data were evaluated considering that aggregate exposure was assessed, i.e., exposure from several sources and pathways of exposure was added up. Since addition of upper percentile values from each of these pathways would result in gross overestimates, mean consumption data were used in the evaluation. The use of mean values is also meaningful because upper percentiles reported in the Comprehensive Database are not robust for several food items due to the small number of actual consu mers in each survey. Overall, the evaluation therefore applies the following approach:

- For each population group and MS, the combined food/drinking water consumption is calculated by adding up the
  ∘ Mean consumption of all canned food items (sum of mean values for several items);
  ∘ Mean consumption of soft drinks;
  ∘ Mean consumption of drinking water.
- For each population group, the maximum combined food/drinking water consumption in any EU Member State is identified (Table S1 in the SMF).
- The values for (a) food consumption (canned food and soft drinks) and drinking water consumption associated with this maximum combined estimate across the EU is taken as an input to estimate exposure.

This approach ensures adequate consideration of potential combined oral exposure from food and drinking water without adding up, e.g., high oral intake via drinking water from one country and high oral intake via canned food from another country. Table 3 summarizes the consumption data for the exposure and risk assessment (see Section S1.2.2 in the SMF for a detailed discussion of these input values).

These data directly reflect the higher consumption of younger age groups per kg of body weight during periods of rapid growth. Combined consumption by adolescents and the very elderly is somewhat overestimated due to overestimated consumption of canned food items in these groups. While mean consumption is used in the exposure assessment, the evaluation includes some conservative elements, e.g., by assuming that all canned/jarred food items and soft drinks are indeed in cans (also see discussion in Section 3.4.3 below and in the SMF, Section S1.2.2 for details).

#### 2.4.2. Textiles

##### Concentration of Hostanox^®^ O 3 in Textiles

Hostanox^®^ O 3 is one of three additives used in combination to provide elastane fibres (often called spandex in North America) with high resistance to degradation by chlorine and atmospheric fumes (cf. European Patent EP1401946B1, https://worldwide.espacenet.com/patent/search/family/023139995/publication/EP1401946B1?q=EP%201401946B1 (accessed on 18 November 2025). While this patent claims concentrations of 0.15–0.3%, company information with respect to the market sector and the specific functionality of the substance in this application indicates that the Hostanox^®^ O 3 concentration in elastane can potentially be in the range of up to 0.5–1.5%. Textiles containing elastane are mixed fabrics, and the elastane fraction in such textiles depends on the fabric and specific application. For cotton and wool, the fraction is typically 1–5%, while it is up to 20% in polyester and polyamide fabrics [21]. Since the upper end value is based on a single source, and higher fractions cannot be excluded, a fraction of 30% is used to calculate the concentration of Hostanox^®^ O 3 in textiles of 0.15–0.45%. The exposure assessment described in the next section uses a mean concentration of 0.30% (calculated mean of the minimum and maximum of the range) and a maximum concentration of 0.45%.

##### Additional Input Data for the Exposure Assessment

The exposure assessment for textiles is based on the amount of textile in contact with the skin and the migration of the substance from the textile. The German Federal Institute for Risk Assessment (Bundesinstitut für Risikobewertung, BfR) provides relevant default values to estimate exposure to textile additives [13]. The default values for skin surface area and body weight, however, solely relate to adults. To cover all seven population groups, an alternative approach was chosen that first estimates the amount of textile in contact with the skin and subsequently considers the Hostanox^®^ concentration in textiles as well as the migration of the substance from textiles. Again, the SMF (Section S1.3) provides more detailed information and justification of the approach.

The amount of textile in contact with the skin is calculated from the total skin surface area. Since the skin surface area and the body weight rapidly change with age, with both changes being related, the total skin surface area normalized to body weight (SA/BW, expressed in m^2^/kg bw), as reported in US EPA’s Exposure Factors Handbook (EFH) [22], is used as a starting point. This approach was chosen since treating skin surface area and body weight as independent may result in unrealistic body types. For example, Phillips et al. [23] demonstrated that combining a 90th percentile value for the skin surface area with a default body weight of 70 kg for adults, as often used in risk assessments, implies a height of the individuals of 264 cm (men) and 205 cm (women). The EFH [22] provides the following mean total SA/BW ratios taken from earlier work by Phillips et al. [23]:

- Infants/toddlers (0–2 years of age): 0.064 m^2^/kg bw;
- Children (2.1–17.9 years of age): 0.042 m^2^/kg bw;
- Adults (18 years or older): 0.028 m^2^/kg bw.

The available SA/BW ratios are not differentiated to the same extent as the population groups defined by EFSA. Based on the age ranges associated with these SA/BW ratios, the highest SA/BW ratio is assigned to the EFSA population groups of infants and toddlers (up to 35 months of age; Table 2), the medium SA/BW is assigned to ‘other children’ and adolescents (3–17 years of age; Table 2) and the lowest SA/BW is assigned to all adult population groups (18 years and older).

It is evident that only part of the total skin surface is covered by textiles. The exposure assessment assumes that 60% of the total skin surface is covered by textiles based on the following considerations. First, this fraction results from the BfR default values for skin surface area in contact with textile of 1 m^2^ and the body weight of 60 kg for adults [13]. These default values result in an SA/BW ratio with textile contact of 0.0167 m^2^/kg, which represents 60% of the total SA/BW of 0.028 m^2^/kg bw used in the exposure assessment. Second, a fraction of 60% approximately corresponds to the skin surface of the entire trunk, one half of the arms plus one half of the legs based on the recommended values for the mean skin surface area of body parts in the US EPA’s EFH [22]. This is considered a reasonable assumption for textiles containing the high elastance fraction of 30% (e.g., bathing suits and other sportswear). The SA/BW ratio in contact with textiles (‘contact SA/BW’ below) is calculated by multiplying the total SA/BW ratio for the different age groups by the fraction of 60%.

In order to calculate the amount of textile in contact with the skin, the contact SA/BW is multiplied by the specific textile weight (expressed in g/m^2^), for which the BfR approach defines a default value of 100 g/m^2^ [13]. This calculation results in the amount of textile in contact with the skin, again normalized to body weight (i.e., g textile/kg bw; see Table S4 in the SMF for specific values). The amount of textile (normalized to body weight) is multiplied by the concentration of Hostanox^®^ O 3 of 0.3% and 0.45%, respectively, which results in the mean and maximum amount of Hostanox^®^ O 3 present in the textile that is in contact with the skin, again normalized to body weight.

Only the fraction of the substance that migrates from the textile will be available for exposure. This fraction is expected to be comparatively low, since substantial migration would result in loss of functionality. The BfR approach defines default worst-case migration rates depending on substance type of 0.5% for textile dyes, 0.2% for hydrophilic textile auxiliaries and 0.1% for hydrophobic textile auxiliaries [13]. Since Hostanox^®^ O 3 is not a textile dye and based on its high log K_ow_ (Table 1), the migration rate of 0.1% is used in the exposure assessment. Therefore, the calculated amount of Hostanox^®^ O 3 present in textiles in contact with the skin is multiplied by 0.1%, resulting in the final external dermal exposure estimate. The calculated exposure is independent of the duration, i.e., identical exposures are estimated irrespective of whether textiles are worn for 15 min or 12 h a day, and the final result is expressed in mg/(kg bw × d).

While the approach to exposure assessment described above uses somewhat ‘atypical’ exposure metrics due to its normalization to body weight, it provides a unique approach that prevents combining unrealistic combinations of body weights and skin surface areas. It can be applied to all population groups covered by this assessment, although with limited differentiation in younger population groups. Nonetheless, it adequately covers the higher SA/BW ratio of children that, as shown below, results in higher estimated dermal exposure of the very young.

Textile parts containing elastane are also used for the leg bands of nappies. Since these leg bands will only cover a small fraction of the skin surface area, the approach described above (assuming 60% of the total skin surface area being covered by textile) covers the assessment of this specific use. To cross-check this suggestion, an alternative exposure assessment approach for nappies is included in Section S2.2 of the SMF and briefly discussed in the Results Section below.

#### 2.4.3. Consumer Use of Sealants

Hostanox^®^ O3 is contained in additives that are used in silyl-modified sealants (SMP) for exterior joints in building and construction, automotive and components applications. SMP sealants are mainly industrial products that are not typically used by consumers, but Hostanox O3 may sometimes be contained in products intended for consumer use. Sea lants and adhesives used by consumers typically involve small product amounts during in frequent DIY projects. The likely exposure scenario resulting from the use of a joint sea lant is modelled in the main assessment. Two further, less likely, exposure scenarios were also modelled: carpet glue for DIY and bottled creative glues. To address un cer tain ties related to the product amount, a consumer exposure scenario from the use of a carpet glue was assessed. Both joint sealants and carpet glues are assumed to be used by adults only and are also assumed to be used infrequently by consumers. To model a theoretical extreme exposure scenario, potential exposure to Hostanox^®^ O 3 from a bottled adhesive that might be used by children was also assessed, since it covers a potentially more vulnerable population group, and exposure might, in this scenario, occur more frequently than in occasional uses of DIY products by adults. It must be stressed that the use of the substance in carpet glue and bottled glue is not confirmed. Evaluations of these additional products are therefore not part of the main assessment but only intend to address the uncertainty in the identification of consumer uses and to assess whether such uses would be a reason for concern.

##### Concentration of Hostanox^®^ O 3 in Consumer Products

The concentration in end-use products was derived from in-house market sector knowledge available for both the Hostanox^®^ O 3 concentration in the additive mixture and the concentration of this additive mixture in end-use products. Based on this information, the Hostanox^®^ O 3 concentration in end-use products ranges between 0.05% and 0.12%, with a typical concentration of 0.08%. However, in-house market sector knowledge also indicates that specialized sealants exist with a higher concentration of 0.28% in the end-use product.

Based on the compiled information, a mean concentration of 0.08% and a maximum concentration of 0.28% is used for the exposure assessment.

##### Additional Input Data for the Exposure Assessment

Inhalation and dermal exposure resulting from the use of Hostanox^®^ O 3 in sealants was modelled with the ConsExpo Web tool (version 1.1.1, January 2023; https://consexpoweb.nl/ (accessed on 14 February 2024); referred to as ‘ConsExpo’ hereafter), developed and maintained by the Dutch National Institute for Public Health and the Environment (Rijksinstituut voor Volksgezondheid en Milieu, RIVM).

All input data used for modelling are based on the parameter values defined in the ConsExpo fact sheet for Do-It-Yourself (DIY) products [24] with body weights adapted to be consistent and conservative (see below). The main assessment considers a joint sealant used by adults three times a year in a bathroom (small room volume of 10 m^3^, but substantial ventilation) covering a moderate amount of product per use (350 g).

The additional assessments consider

- A carpet glue as another DIY product used by adults once a year on a large surface (larger room volume of 58 m^3^, limited ventilation) covering a high product amount per use (14.3 kg);
- A bottled glue used by children in a regular room (20 m^3^, limited ventilation) involving a product amount of 10 g per event.

These products and scenarios cover a rather broad range of use conditions and therefore appear adequate for the exposure and risk assessment. While the use in carpet glues appears unlikely, since such products apparently do not require light stabilization, this product was included to cover products applied in large quantities.

In addition to the parameter values provided in the ConsExpo fact sheet, this source also defines the scenario settings, e.g., the exposure model. For example, inhalation exposure due to evaporation of the substance is modelled by default for the products assessed here (see Section S1.4 in the SMF).

The exposure on the day of use estimated with ConsExpo was used as a starting point for the risk assessment. It is, however, evident that exposure on the day of use is inadequate for comparison with long-term DNELs for joint sealants and carpet glues that are used only rarely by consumers according to the ConsExpo fact sheet [24]. The relevant ECHA Guidance [12] outlines a refinement approach for such situations. Infrequent uses are characterized as involving ‘not more than 15 days of exposure occur per year’ [12], which applies to these two DIY products as shown above. In such a case, the relevant ECHA Guidance foresees that ‘the assessment factor of 6 for extrapolation from a subacute study to the long-term consumer DNEL can be omitted’ [12]. Based on this refinement approach, the dermal DNEL for the risk assessment of both DIY products is increased from 0.17 to 1 mg/(kg bw × d). Since inhalation exposure during the use of these DIY products is negligible (see Results Section below), this refinement is only applied to dermal exposure.

For inhalation exposure, the mean concentration on the day of exposure is used for risk assessment. Averaging over prolonged periods of time (e.g., annual average concentration in air) is not applied, since modelled inhalation exposure was negligible for all three products. The higher peak concentration in air and mean event concentration (both estimated for the use phase) are not used, since they exist only for short periods of time and are inadequate for comparison with the long-term inhalation DNEL. Nonetheless, all these exposure estimates are provided together with all relevant input data in the ConsExpo report (see SMF, Section S1.4).

To address potential exposure of children, the use of ‘bottled glue: universal/wood glue’ (as defined in ConsExpo) was modelled with the default settings defined in the ConsExpo fact sheet [24]. The input values (e.g., for product amount and use frequency) in the fact sheet relate to the use of a wood glue by adults. To adapt this scenario to the use by children, the following approach was used. For a conservative estimate, exposure of kindergarten children was assessed: from the population groups available in the population databases embedded in the ConsExpo Web tool, children 2–3 years of age with a body weight of 12.4 kg were selected. This body weight represents the 25th percentile and is recommended in ConsExpo as the worst-case default value for this age group [17]. This age group is practically identical to the toddlers defined by EFSA (see Table 2), and the exposure estimate was therefore assigned to this population group. All other input values from the fact sheet were retained. For example, the product amount of 10 g of glue (relating to gluing together two slides of wood by adults) was not changed, although this may be considered a very high value for use by toddlers. Also, the use frequency of 36 applications per year was retained, since it does not have an impact on the exposure estimate in our approach, which uses exposure levels on the day of exposure without correction for infrequent use of bottled glue (in contrast to the DIY products). Therefore, dermal exposure on the day of exposure resulting from the use of bottled glue by children is compared with the dermal DNEL of 0.17 mg/(kg bw × d). This approach is conservative, since it implies daily use of a relatively large amount of glue by toddlers.

As discussed in Section S1.4 in the SMF, the body weight for adults used in ConsExpo modelling represents the (rounded) 25th percentile for females from the ConsExpo fact sheet [17] to cover the use of products by females. Conservative assumptions for the body weight (i.e., 25th percentile values) for toddlers and adults were chosen, since the conservatism of several other input parameters for ConsExpo modelling is unknown.

#### 2.4.4. Calculation of Aggregate Exposure and Risk

Exposure and risk assessments are often performed in a tiered way, in which conservative assumptions are used at the lowest tier (see e.g., [12]). The degree of conservatism is not always readily apparent and may sometimes represent worst-case assumptions (i.e., maximum exposure estimates) or very high percentiles (e.g., 95th percentiles). The main purpose of such approaches is to identify the need for refinement. If safe use can be demonstrated based on such lower tier approaches applying conservative assumptions, no need for refinement exists.

In aggregate exposure assessments addressing simultaneous exposure from several sources, adding up maximum or upper percentile exposure estimates runs the risk of gross overestimates for statistical reasons alone. Therefore, our aggregate exposure assessment applies the following principles:

- Mean and the maximum Hostanox^®^ O 3 concentrations as derived above for the specific products are used to estimate mean and maximum exposure to the substance.
- Mean input values are generally used for all other parameters determining the exposure estimate. For all uses, some conservative elements are included in the selection of input values. This primarily applies to input values (a) which cannot be further differentiated (e.g., all canned/jarred food items assumed to be in cans), (b) for which no robust mean can be derived (e.g., elastane fraction in textiles) and (c) which are based on default values provided by established methodologies (e.g., the default migration rate for textiles).
- The mean exposure is added up in an aggregate exposure assessment that considers all pathways of exposure.
- Maximum exposure estimates are provided but are not added up in the aggregate exposure and risk assessment.

As noted above, the calculated exposure will be compared with the long-term DNELs derived for Hostanox^®^ O 3. Dividing the exposure by the DNEL results in the Risk Cha rac terization Ratio (RCR), which is calculated for each exposure route (e.g., oral for expo sure via food and dermal for exposure via textiles). For the relevant consumer products, dermal and inhalation RCRs are calculated and added up, but the results below demon strate that inhalation exposure and risk are negligible. The RCR represents the risk metric applied in chemical safety assessments conducted under the REACH Regulation, while risk is expressed differently in other regulatory areas [25]. We chose the RCR as a uniform risk metric to allow for aggregation of risks from all uses irrespective of the regulatory area. The risk from a substance is considered controlled if the RCR is below 1 [12].

## 3. Results

### 3.1. Exposure and Risk from Food and Drinking Water

Oral exposure to Hostanox^®^ O 3 (a) from canned food and soft drinks as well as (b) from drinking water is assessed based on the input data discussed above. Table 4 provides the oral exposure estimates and the resulting oral RCR values covering combined oral exposure from canned food, soft drinks and drinking water based on mean Hostanox^®^ O 3 concentrations. The SMF (Tables S1, S2 and S8) provides detailed data for each of the three food categories and the population groups, also including values resulting from the application of maximum Hostanox^®^ O 3 concentrations.

These data show that the combined oral exposure from all sources based on mean Hostanox^®^ O 3 concentrations is well below the DNEL of 0.17 mg/(kg bw × d), resulting in oral RCR values below 0.08 for all population groups. It is evident that oral exposure is also well below the TDI of 0.1 mg/(kg bw × d). As shown in Table S8 of the SMF, the maximum exposure estimates for each of the three categories are also well below these reference values (maximum: 0.0161 mg/(kg bw × d) for exposure of infants via drinking water).

While the calculated risks are very low for all population groups, the highest RCRs are observed for small children and decrease with increasing age. This finding was expected since food consumption—when normalized to body weight—decreases with age (Table 3). The findings therefore demonstrate the adequacy of using food consumption data normalized to body weight.

Alcoholic beverages in cans were not considered in the exposure and risk assessment for several reasons. First, all soft drinks consumed were assumed to be in cans in the expo sure assessment for this category, since the Comprehensive Database does not allow for further differentiation. This approach introduces a conservative element with respect to the consumption data for soft drinks. Second, the Comprehensive Database also does not allow for a differentiation by the type of packaging for alcoholic beverages. Third, the ad dition of another category of liquid products would likely result in the unrealistic ad di tion of consumption data, since those consuming alcoholic beverages may be less likely to consume soft drinks and drinking water on the same day at the levels derived above.

In addition, it cannot be completely ruled out that some canned food items may not be reported (or underreported) in the Comprehensive Database. For example, the data show a large variability in the number of canned food items per population group and country (Table S2 in the SMF).

We therefore conducted additional analyses that compare our food and drinking water consumption values with data established by EFSA. These analyses demonstrate that our approach is unlikely to underestimate mean consumption of canned food, soft drinks and drinking water (SMF, Section S1.2.2).

### 3.2. Exposure and Risk from Textiles

Dermal exposure from the use of Hostanox^®^ O 3 in textiles is assessed based on the input data discussed above. Table 5 summarizes the mean and maximum exposure estimates for textiles and the resulting RCRs.

The assessment shows low dermal exposure and dermal RCRs below 0.07 when the mean Hostanox^®^ O 3 concentration is used. A maximum RCR of about 0.1 for infants and toddlers results from using the maximum Hostanox^®^ O 3 concentration in food and drin king water. Dermal exposure and RCRs decrease with age, a finding resulting from the decrease of the SA/BW ratio with age. The finding of identical exposure estimates for (a) infants and toddlers; (b) other children and adolescents; and (c) adults, elderly and very elderly directly results from the fact that skin surface areas normalized to body weight are only available for three age groups and therefore had to be assigned to the seven population groups in a meaningful way (see above). Nonetheless, the general trend reflects the higher exposure of the very young and therefore adequately reflects anthropometric characteristics. It may be questioned whether infants and toddlers (up to 35 months of age; see Table 2) do in fact wear garments with a high elastane concentration of 30% and covering 60% of their skin surface, as assumed in this assessment. Such assumptions may primarily apply to bathing suits and other sportswear unlikely to be worn by infants and toddlers.

However, infants and toddlers may be exposed to Hostanox^®^ O 3 from elastane fibres used in leg bands of nappies. While we expected this application to be covered by the main exposure assessment due to the large skin surface area assumed to be in contact with the skin, we evaluated potential Hostanox^®^ exposure from nappies with an alternative approach. The corresponding exposure assessment is largely based on input data from a report by the French Agency for Food, Environmental and Occupational Health & Safety (Agence nationale de sécurité sanitaire de l’alimentation, de l’environnement et du travail, ANSES) [26] using the mean and maximum Hostanox^®^ O 3 concentrations in textiles derived above and additional data on body weights of the very young [27,28]. This alter na tive approach is based on nappy weights and use frequencies and largely independent of assumptions on the skin surface area. The alternative approach results in dermal expo sure estimates and dermal RCRs that are about five times lower than the ones obtained in the main assessment (see Section S2.2 in the SMF for details). Since identical Hostanox^®^ O 3 concentrations and identical migration rates were assumed in both approaches, the difference is due to differences in estimating the amount of textile in contact with the skin. A much larger difference may be expected based on the substantial difference in the skin surface area exposed (nappy leg bands vs. 60% of the total skin surface). However, the main conclusion that the exposure and risk assessment for textiles shown above covers the use of elastane in the leg bands of nappies remains valid. Further discussion and refinement of the exposure assessment approach is therefore not necessary.

The estimated exposure from textiles is low, partly because a low migration rate of 0.1% from textiles was assumed. We consider this low migration rate justified for several reasons. First, this value represents the default ‘worst-case’ migration rate for hydrophobic textile auxiliaries [13]. Second, Hostanox^®^ O 3 must remain in the garment to exert its functionality of protecting elastane fibres, and a low migration is therefore essential. Finally, the exposure assessment assumes that the Hostanox^®^ O 3 concentration remains unchanged over prolonged periods of time. If a substantially higher migration rate, e.g., of 2.5%, is assumed, only 50% of the initial amount will be present in the garment after 4 weeks, and the substance will have essentially vanished from the garment after 1 year (<0.01% of the initial amount). Under such conditions, a comparison with the long-term DNEL is inadequate. In contrast, about 70% and 50% of the substance is estimated to be present in the garment after about 1 and 2 years, respectively, with the low migration rate in our approach.

### 3.3. Exposure and Risk from Consumer Uses

Inhalation and dermal exposure from the use of Hostanox^®^ O 3 in sealants is assessed based on the input data discussed above. Table 6 summarizes the mean and maximum exposure estimates for this DIY product as well as the associated RCRs.

In line with the low vapor pressure of Hostanox^®^ O 3, inhalation exposure is negli gi b le. Only evaporation of the substance from this DIY product was modelled in ConsExpo based on default settings (see Section S1.4 in the SMF). Aerosol formation during use (as another mechanism of release to air) was not considered in modelling since the potential for aerosol generation is considered low (if any) for this use. This statement only applies to this specific product but would not be valid for spray applications. As a result of negligible inhalation exposure, the combined RCRs are identical to the dermal RCRs.

The combined RCRs are below 0.02 when the mean Hostanox^®^ O 3 concentration is used and below 0.7 when the maximum Hostanox^®^ O 3 in sealants is used for the exposure assessment.

Similar findings are noted for the two additional products assessed (carpet glue used by adults and bottled glue used by toddlers). Inhalation exposure is negligible for both products, and the following discussion therefore focusses on dermal exposure. For carpet glues, the RCRs after correction for infrequent uses (see Material and Methods above) are 1.8 times higher than for the joint sealant (mean 0.0332, maximum: 0.116). While a higher contact rate is assumed in ConsExpo for the joint sealant (50 mg product/min) than for the carpet glue (30 mg product/min), the release duration is assumed to be considerably higher for the carpet glue (90 min) than for the joint sealant (30 min). The released amount of 2700 mg carpet glue is 1.8 times higher than the released amount of joint sealant (1500 mg). The observed differences are therefore a direct result of the default values in the ConsExpo fact sheet for DIY products [24]. The potential use of bottled glue by toddlers results in similar RCRs to those modelled for the use of carpet glues by adults (mean 0.0306, maximum: 0.106). However, the RCRs for bottled glue used by toddlers reflect more conservative estimates than the ones for the two DIY products, since the exposure estimates do not consider the infrequent (i.e., less than daily) use of such products. SMF Sections S1.4 and S2.3 provide detailed input and output data for ConsExpo modelling as well as the resulting mean and maximum RCRs.

The results demonstrate that even the daily use of adhesives containing the maximum concentration of Hostanox^®^ O 3 by toddlers does not present a cause for concern based on ConsExpo modelling.

### 3.4. Aggregate Exposure and Risk from All Sources

#### 3.4.1. Main Findings

**Figure 1:**
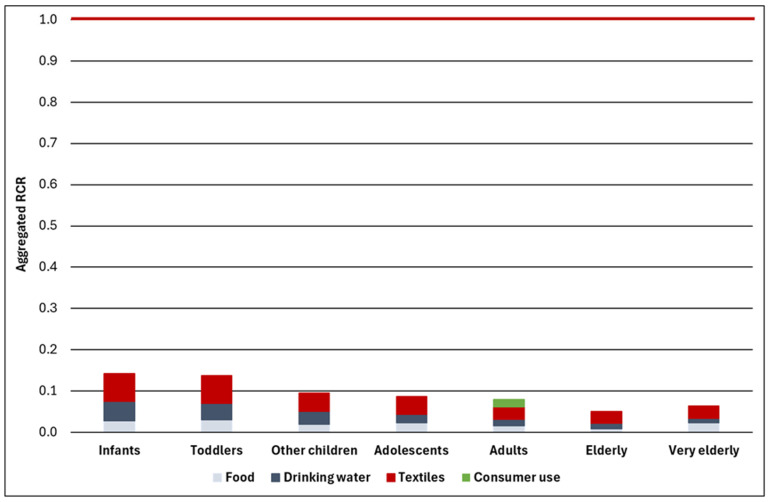
Mean pathway-specific and aggregate RCRs of the main assessment. The red line indicates the RCR of 1, below which risks are controlled, and the *Y*-axis is deliberately set to an RCR of one to illustrate the large margin (see table below for specific RCRs).

Figure 1 and Table 7 show the RCRs differentiated by population group and pathway of exposure as well as the aggregated RCRs based on mean Hostanox^®^ O 3 concentrations. The presentation of RCRs (rather than exposure) is meaningful, since different routes of exposure and exposure metrics are concerned (oral, dermal and/or inhalation). Figure 1 shows the RCRs for the main assessment only. Table 7 provides the RCRs of the main assessment (set in bold) as well as the RCRs for the additional, unconfirmed consumer uses (set in italics).

The main aggregate exposure and risk assessment shows low aggregate RCRs < 0.1 for all population groups except infants and toddlers. The somewhat higher aggregate RCRs of about 0.14 for infants and toddlers reflect (a) the higher food intake per kg body weight of these groups (Table 3) due to the ‘higher energy requirement during rapid growth’ [29] and (b) their higher skin surface area per kg body weight (see Additional Input Data for the Exposure Assessment in Section 2.4.2) characteristic for these age groups [22,23,30].

Consideration of the unconfirmed use of bottle glue by toddlers and carpet glue by adults increases the aggregate RCRs, but the resulting values of 0.168 (toddlers) and 0.113 (adults) are still well below 1.

Collectively, the aggregate exposure and risk assessment demonstrates low risks even when exposure from several sources is considered. This overall conclusion is valid even after inclusion of unconfirmed consumer uses.

#### 3.4.2. Additional Findings

Since the exposure assessment for food, drinking water and textiles is based on anthropometric input values that are normalized to body weight, the aggregate exposure and risk assessment is considered an adequate reflection of a potentially slightly higher exposure and risk of the very young due to specific anthropometric differences. Since (small) children may be more vulnerable to the effects of chemicals, their coverage in exposure assessments is crucial. Due to anthropometric differences (including age-specific differences in food consumption), the pathway-specific as well as the aggregate RCRs decrease with age with only minor deviations from this general trend in some cases. For example, the RCRs from food for adolescents and the very elderly deviate from this general trend. However, the oral exposure estimates for these two population groups are considered overestimates, since they involve some double counting of canned food items. Since RCRs for these population groups are very low, no attempt was made to refine the corresponding exposure (see SMF, Section S1.2.2, for details). However, the RCRs provided for adolescents and the very elderly should be treated with caution and are not further addressed in the discussion below.

Interestingly, the data show a lower risk from exposure via food for the elderly, as another potentially more vulnerable population group. This lower exposure directly results from the lower consumption of canned food, soft drinks and drinking water by this population group (see Table 3). Since the RCRs from exposure via textiles is identical for adults and the elderly, the aggregate RCR for the elderly of 0.0506 is lower than the one for adults (0.0611 after subtraction of the RCR for consumer use).

The contribution of the different pathways of exposure to the aggregate RCR is similar across population groups, with oral exposure (food and drinking water combined) and dermal exposure via textiles each contributing about 50% to the aggregate risk (see SMF, Section S2.4).

These additional findings (a) illustrate the importance of assessing exposure of different population groups and (b) demonstrate the benefits of using defined population groups, ideally with input data normalized to body weight for each population group.

#### 3.4.3. Conservativeness of the Aggregate Exposure and Risk Assessment

The aggregate exposure and risk assessment presented above is based on mean Hostanox^®^ O 3 concentrations and mean values for some of the other input parameters (e.g., food and drinking water consumption and SA/BW ratio; Table 8). However, more conservative values are assumed for other input data (e.g., with respect to consumption of canned food and soft drinks; Table 8). For example, the fraction of consumers of soft drinks is very low for infants and toddlers (<1% and <10%, respectively; see Section S1.2.2 of the SMF), and the mean consumption in these cases—together with the assumption that all soft drinks are in cans—may result in gross overestimates of exposure.

In addition to the conservative assumptions shown in Table 8, the aggregate assessment furthermore assumes that a person is exposed to Hostanox^®^ O 3 via all pathways on every single day of the year. For example, the exposure assessment assumes that an infant up to 11 months of age with a body weight of 5 kg on every single day

- Consumes 37 g of canned food with all canned/jarred food items being assumed to be in cans (rather than jars);
- Consumes 80 mL of soft drinks containing Hostanox^®^ O 3 with all soft drinks assumed to be in cans;
- Drinks 270 mL of tap water containing Hostanox^®^ O 3;
- Wears garments that cover 60% of the skin surface and are made of fabric with 30% elastane that contains Hostanox^®^ O 3.

The assumption of concurrent exposure via all pathways is certainly conservative for all pathways except exposure via drinking water. It was primarily implemented since no robust information on the frequency of use (or consumption of specific food items) is available.

For the consumer uses, we applied conservative assumptions for body weights, since the conservatism of several other input data is unknown.

Based on these considerations, the aggregate assessment based on mean Hostanox^®^ O 3 concentration results in risk estimates that are higher than a ‘mean risk’. While the protection level associated with this assessment cannot be quantified, the low RCRs even in the aggregate risk assessment therefore demonstrate the safe use of the substance in the above applications. As noted above, this conclusion remains valid if unconfirmed con sumer uses by toddlers and adults are included.

Furthermore, even the aggregate RCRs based on maximum Hostanox^®^ con cen tra tions in all products and considering all consumer uses (including unconfirmed ones) is below 0.4 for all population groups. Since adding up maximum pathway-specific RCRs in an aggregate assessment is considered inappropriate (see Section 2), the corresponding calculations of aggregate RCRs based on maximum Hostanox^®^ concentrations are not provided. Nonetheless, this finding supports the general conclusions made.

Overall, the aggregate exposure and risk assessment demonstrates low risks and the safe use of Hostanox^®^ O 3 in the applications covered.

## 4. Discussion

This study presents an aggregate exposure and risk assessment that—to our knowledge—is unique in several respects. First, it integrates exposure and risk from diffe rent applications of the substance that are typically assessed separately. Second, this as sess ment includes seven population groups aligned with EFSA’s methodology that are not assessed, e.g., under the REACH Regulation. The approach ensures consideration of population groups generally assumed to be potentially more vulnerable and—as shown in this study—who may experience higher exposures (i.e., infants and toddlers). Third, this study used key input values normalized to body weight for all exposure pathways except consumer uses, preventing unrealistic combinations of values for dependent pa ra meters (e.g., body weight and skin surface area). Finally, the approach therefore adequately reflects the characteristic higher food intake and skin surface area of the very young, when normalized to body weight.

The methodology described in this study was developed for application in practice also considering future regulatory developments in the EU as discussed in Section 4.3 be low. In addition, all calculations are simple to apply, and all inputs and outputs are trans parent and well documented. This not only allows application, e.g., by industry stake holders, but also facilitates regulatory scrutiny by authorities. The methodology is based on external exposure estimates that are compared with external reference values (i.e., DNELs). This approach ensures that the methodology can be applied to many other sub stances, since oral, inhalation and dermal DNELs are available for a comparatively large number of substances. In contrast, other reference values (e.g., ADIs and TDIs) are only available for a comparatively small number of substances and only cover oral exposure. Furthermore, using reference values derived in the context of EU legislation may increase regulatory acceptance of such assessments. Finally, pathway-specific exposure and risk is assessed separately before aggregation, which allows for evaluating pathway con tri bu tions (also see Section 4.3 below).

The approach developed in this paper differs from other aggregate exposure assess ment approaches. In fact, we were unable to locate publications that address the exposure pathways covered by this study in an aggregate consumer exposure assessment. Other aggregate exposure assessment approaches include, e.g., high-throughput exposure pre dictions [31,32], which are primarily developed for prioritization purposes but are un likely to be useful for substance-specific assessments. The development of aggregate ex posure pathways for combination with adverse outcome pathways [33,34] focusses on inter nal (biomarker) exposure metrics that can be compared with internal toxicity metrics. In practical application of such approaches to specific substances [35,36], no reference values are applied, and exposure via different pathways is not evident.

### 4.1. Data Accessibility and Challenges

The applied methodology demonstrates the general feasibility of an aggregate consumer exposure and risk assessment, but such assessments encounter several challenges. The concentration of the substance assessed needs to be known for the different applications in which it is used. As shown by this case study, concentrations in food and drinking water may be based on legal limit values (e.g., MTCtap) or migration studies (e.g., for use in FCM) required under the respective legislation. Other appropriate data or justified assumptions (e.g., by considering the water solubility of the substance) may inform the discussion of this key input value. Exposure resulting from the use in textiles or due to consumer uses of mixtures containing the substance, in contrast, require detailed knowledge to derive realistic mean and maximum values. Such knowledge may be available to REACH registrants in some cases based on their sector- or application-specific knowledge, including an appreciation of the functional requirements of the substance in specific uses. In this case study, such knowledge was available, but this may not always be the case. Default assumptions representing very high concentrations are unlikely to be helpful in such situations for most substances, since they may result in very high exposures exceeding the reference values. Overall, the concentration of the substance in relevant end products (Table 9) may need to be based on non-public data for several uses.

Apart from the concentration of the substance in the products, challenges also relate to the generation of additional input data for the exposure assessment. Food and drinking water consumption data for Europe are publicly available in the Comprehensive Database (Table 9). Using the Comprehensive Database has several advantages. First, information is typically available for the seven population groups defined by EFSA. Second, values normalized to body weight are available, the use of which prevents inappropriate combinations of per capita consumption and body weight data. Finally, information from most EU Member States is available (Table S1 in the SMF), which allows for covering differences in consumption patterns across the EU in the exposure assessment. The main challenge lies in the extraction and evaluation of the consumption data for specific food items or categories of food items in the context of an aggregate exposure assessment. The detailed description in Section S1.2.2 of the SMF illustrates the complexities involved and some of the problems encountered. In addition, the approach applied in this study for the extraction and evaluation of such data is not by any means harmonized.

For dermal exposure to the substance from textiles, information on the SA/BW ratio as a key input parameter is publicly available (Table 9). One of the problems encountered in this study is the fact that the SA/BW ratios used [22,23] are only available for three rather broad age groups that had to be assigned to the seven EFSA population groups. While future research may develop SA/BW ratios with a higher degree of differentiation for subjects 3–17 years of age (‘other children’ and ‘adolescents’ in EFSA’s population group system; Table 2), the results from the case study reflect the key difference between the very young and adult population with respect to dermal exposure.

The assessment of exposure resulting from consumer uses (application of sealants and other products) did not encounter major challenges since the established ConsExpo Web tool could be used for this purpose, and approaches for considering the infrequent nature of the tasks is established [12]. Furthermore, the ConsExpo Web tool is freely available, and all input data are published in ConsExpo fact sheets, publicly available also for other consumer products (see SMF, Section S1.4).

### 4.2. Uncertainties of Key Input Data

For the substance-specific assessment in this study, knowledge of the substance concentration was available based on marketing expert knowledge. While these data represent our best current understanding and are considered reliable estimates, some degree of uncertainty remains inherent in real-world application scenarios. The substance concentration may represent a more significant source of uncertainty in other assessment cases where such detailed application information is not available.

The food consumption values applied in this study involve some uncertainty, pri marily due to the use of the substance in cans, i.e., a rather specific FCM (see extended dis cussion in the SMF, Section S1.2.2). For this reason, some conservative assumptions were made for deriving the consumption data (Table 8). Nonetheless, aggregate assessments would benefit from harmonized consumption data—to be derived from the Com pre hen sive Database—for food items relevant for FCM, ideally differentiated by the type of FCM.

For dermal exposure from textiles, SA/BW ratios are considered robust but lack sufficient age differentiation (see above). The assumption of 60% of the total skin surface being in contact with textiles is considered conservative for an aggregate assessment, although it does not cover all kinds of textiles. Also, the migration rate is based on a ‘worst case’ default of the BfR methodology adding to the conservative assumptions (Table 8). While the low migration rate applied (0.1%; Section 2.4.2, Additional Input Data for the Exposure Assessment) substantially reduces daily dermal exposure, it is adequate for the modelling approach, which assumes that the substance concentration in the textile does not substantially change over time. In contrast, a migration rate of 1% would result in only one half of the initial amount of the substance being present in the textile after 70 days. In such a situation, the dermal exposure should not be compared with a chronic (i.e., lifetime) reference value.

For consumer uses, the main uncertainty relates to the amount of product in contact with the skin that is assumed in ConsExpo. This amount is calculated by multiplying the contact rate (in mg/min) with the duration (in min). While the ConsExpo default values for these parameters are based on expert judgement, they are discussed in some detail in the fact sheet [24]. A critical appraisal of these default values is outside the scope of this article.

To address the potential uncertainty of this deterministic approach, we have not only applied some conservative assumptions (Table 8) but have also calculated exposure assuming maximum concentrations of the substance. While the findings from these additional calculations increase the confidence in the conclusion on substance-specific risks, the uncertainty of the assessment cannot be quantified. A probabilistic exposure assessment using distributions for key input data may be developed in the future and be better suited than combining upper percentile values in a deterministic approach. Such a probabilistic exposure assessment would more fully address the variability and uncertainty in the data and the final exposure estimate. While probabilistic exposure assessments are occasionally performed, e.g., in the context of dietary exposure assessments, see e.g., [37,38], they are the exception rather than the rule and require substantial additional efforts. For such probabilistic assessments to be used in practice, i.e., by industry stakeholders or authorities, the development of a dedicated software would be beneficial. For several key input data of our study (e.g., food and drinking water consumption and SA/BW ratios), descriptive statistics are reported that in principle would allow for defining distributions for use in such software. Pending such a development, upper percentile values could be used for selected input parameters in principle, but this would require careful statistical examination when adding up exposures in an aggregate assessment to prevent unrealistic combinations.

### 4.3. Practical Issues and Regulatory Context

The application of aggregate exposure and risk assessment in a regulatory context encounters substantial challenges, not the least because different uses are covered by legislation within the remit of different European agencies (e.g., ECHA and EFSA). The scientific and regulatory challenges were recently summarized in a report for EFSA [4], and we would like to highlight an additional, more practical aspect. There is no platform or tool for reporting aggregate exposure and risk assessments of the type described in this study. For example, ECHA’s CHESAR tool for assessments performed under the REACH Regulation does not allow us to integrate exposure assessments for uses covered by other regulatory areas.

The envisaged ‘common data platform’ described in the proposed OSOA package [2] also does not contain any provisions in this respect. Concerning consumer exposure, it seems to place a strong emphasis on biomonitoring data. While results from biomonitoring studies do indeed reflect aggregate exposure from all sources, it is generally impossible to identify the sources of exposure and the contribution of different pathways of exposure. Approaches as applied in this study, in contrast, readily identify the source contribution (Figure 1 and Table S11 in the SMF) and therefore provide insights that will be impossible to generate from biomonitoring in most cases. In addition, biomonitoring requires detailed knowledge of the substance (e.g., with respect to toxicokinetic behaviour and identification of relevant metabolites), substantial efforts (e.g., in development and validation of analytical methods) as well as resources to generate representative data. It is therefore hardly conceivable that representative biomonitoring data will be generated for many of the chemicals with potential consumer exposure. For example, the EUPL of substances for drinking water contact materials alone contains more than 2 000 entries, and an MTCtap is provided for more than 500 of these. Finally, it is questionable whether representative biomonitoring data for infants and toddlers will be generated, e.g., due to practical and ethical constraints in sampling these age groups. Thus, a recent review of European biomonitoring studies on phthalates—a substance group very widely studied by biomonitoring—does not contain data for infants and toddlers [39]. The lack of biomonitoring data for these population groups is important given that they may show higher exposures via food and dermal contact due to their specific characteristics, as noted in our case study.

More specifically to the substance assessed, the aggregate exposure and risk assess ment covering uses assessed separately under different legislations by default indicates that the use of Hostanox^®^ O 3 is safe for consumers (RCRs well below 1; see Section 3.4). If for other substances aggregate RCRs above one are obtained, the approach described in this article allows for identifying the uses contributing the highest share to the aggregate RCR. Such findings may, e.g., inform industry to limit the concentration in specific applications or authorities to target regulatory risk management measures.

These substance-specific findings are discussed in the context of the generic approach to risk management that the CSS envisages to extend ‘to ensure that consumer products (…) do not contain chemicals that cause cancers, gene mutations, affect the reproductive or the endocrine system, or are persistent and bioaccumulative’ [1]. Such a generic approach to risk management is based on the hazardous properties of a substance (e.g., its classification according to the European CLP Regulation) on the one hand and generic exposure considerations on the other hand, such as widespread uses of the substance, its use in applications resulting in exposure of vulnerable groups (e.g., children), or situations in which exposure is difficult to control [1,40]. Since generic approaches to risk mana ge ment do not require full-scale exposure and risk assessments, they are typically easier and more rapidly implemented than risk management measures derived from such in-depth assessments. Existing legislation already contains ‘generic’ provisions to limit consumer exposure. For example, a harmonized classification for reproductive toxicity under the CLP Regulation (Repr. 1A and 1 B) with few exceptions triggers bans of the substance as such and in mixtures available to the general public according to entry 30 of Annex XVII to the EU REACH Regulation.

The generic approach to risk management, if implemented as envisaged in the CSS, aims at extending this approach to all consumer products, ‘including, among other things, food contact materials, toys, childcare articles, cosmetics, detergents, furniture and textiles‘ to ensure ‘protection against (the) most harmful chemicals’ [1]. The CSS does not define ‘most harmful chemicals’, but a recent communication from the European Commission [41] defines fines ‘most harmful substances’ as chemicals having the ‘hazard properties’ of CMR substances (Cat. 1A and 1B), endocrine disruptors (Cat. 1), respiratory sensitizers (Cat. 1) or substances causing specific target organ toxicity following repeated exposure (STOT-RE Cat. 1; this list only relates to human health endpoints), apparently relating to classifications according to the CLP Regulation.

This extension of the generic approach to risk management is likely to affect many chemicals. For example, the European Union List of Authorized Substances in plastic FCM (Annex I of Regulation 10/2011/EU, as amended by Regulation (EU) 2024/3190) currently contains almost 1 200 entries. As shown in Section S3 in the SMF, 24% of the substances that (a) are identified by a CAS or EC number and (b) have harmonized classifications in Annex VI to the CLP Regulation meet the criteria for their identification as ‘most harmful substances’ for human health. Most of these chemicals are also included in the EUPL for drinking water contact materials. This fraction is likely an underestimate (see SMF Section S3) and is expected to increase in the future since substances continue to be classified for relevant properties. Hostanox^®^ O 3 is a case in point, if the harmonized classification for reproductive toxicity (Repr. 1B) recommended by ECHA’s RAC is adopted by the European Commission. An increase in this fraction may also result from classifications for the new hazard class of endocrine disruption for human health that is not reflected in the evaluation summarized above (see SMF, Section S3).

It is outside the scope of this study to assess whether all or some of the uses covered by the aggregate exposure and risk assessment, e.g., in FCM and in materials in contact with drinking water, qualify as ‘essential uses’ according to the criteria laid down in the European Commission communication [41]. If not, the use of ‘most harmful substances’ may be subject to the generic approach to risk management with the possible result of such substances being phased out in such applications, even if the exposure and risk associated with such uses is low (as illustrated in our case study). If, in contrast, such uses are considered ‘essential’, they may not become subject to the generic approach to risk management but rather be assessed in more detail. Such an in-depth assessment would require a detailed chemical risk assessment, which—in our opinion—could involve an aggregate exposure and risk assessment. Our case study may inform such an assessment for Hostanox^®^ O 3.

## 5. Conclusions

This study demonstrates the general feasibility of conducting aggregate consumer exposure and risk assessments within an EU regulatory context. It develops a transparent methodological approach that can relatively easily be applied by different stakeholders. This study may contribute to further developing aggregate exposure and risk assessment approaches in the EU. Such a development would benefit from defined, scientifically justified input values agreed upon across regulatory areas, including harmonized food consumption data. In the long term, the approach may incorporate more advanced statistical methods that would allow for a better assessment of variability and uncertainty. The case study demonstrates the safe use of Hostanox^®^ O 3 when aggregating exposure from all known sources.

## Figures and Tables

**Table 1:** Physicochemical properties and toxicological reference values for Hostanox^®^ O 3.

Parameter (Method *)	Value
Physical state	Solid **
Molecular weight	795 g/mol
Vapor pressure (OECD TG 104)	<<1 × 10^−6^ Pa **
Water solubility (OECD TG 105)	0.5 mg/L **
Partition coefficient n-octanol/water (log Kow) (OECD TG 117)	>>7 **
DNEL, general population, long-term inhalation	0.29 mg/m^3^
DNEL, general population, long-term oral exposure	0.17 mg/(kg bw × d)
DNEL, general population, long-term dermal exposure	0.17/1 mg/(kg bw × d) ***

All information based on ECHA CHEM [5]. * OECD Test Guideline (TG) number, according to which testing was performed, given in brackets. ** Value measured at room temperature. *** For infrequent uses of DIY products, a higher DNEL of 1 mg/(kg bw × d) is used (see text for details).

**Table 2:** Population groups covered by the exposure and risk assessment.

Population Group *	Age *
Infants	up to and including 11 months of age
Toddlers	from 12 up to and including 35 months of age
Other children	from 36 months up to and including 9 years of age
Adolescents	from 10 up to and including 17 years of age
Adults	from 18 up to and including 64 years of age
Elderly	from 65 up to and including 74 years of age
Very elderly	from 75 years of age and older

* Based on EFSA [11].

**Table 3:** Food and drinking water consumption (in g/(kg bw × d) used as input for the exposure and risk assessment.

	Infants	Toddlers	Other Children	Adolescents	Adults	Elderly	Very Elderly
Canned food	7.38	9.10	6.94	10.6 *	4.22	3.61	14.6 *
Soft drinks	16.1	16.1	9.60	14.2	9.12	3.89	4.72
Drinking water	53.5	45.9	35.4	12.0	18.1	14.0	13.0

* Overestimated due to double counting (see SMF, Section S1.2.2 for details).

**Table 4:** Exposure and risk assessment: food and drinking water.

Population Group	Combined Oral Exposure (mg/(kg bw × d))	Oral RCR
Infants	0.0127	0.0748
Toddlers	0.0119	0.0701
Other children	0.00862	0.0507
Adolescents	0.00728	0.0428
Adults	0.00538	0.0317
Elderly	0.00360	0.0212
Very elderly	0.00581	0.0342

All values rounded to three significant figures, but unrounded values used for calculation.

**Table 5:** Exposure and risk assessment: textiles.

Population Group	Dermal Exposure (mg/(kg bw × d))		Dermal RCR	
	Mean	Maximum	Mean	Maximum
Infants	0.0114	0.0171	0.0672	0.101
Toddlers	0.0114	0.0171	0.0672	0.101
Other children	0.00750	0.0113	0.0441	0.0662
Adolescents	0.00750	0.0113	0.0441	0.0662
Adults	0.00500	0.00750	0.0294	0.0441
Elderly	0.00500	0.00750	0.0294	0.0441
Very elderly	0.00500	0.00750	0.0294	0.0441

All values rounded to three significant figures, but unrounded values used for calculation.

**Table 6:** Exposure and risk assessment: joint sealant (main assessment).

Exposure Estimate	Inhalation		Dermal		Combined
	Exposure (mg/m^3^)	RCR	Exposure (mg/(kg bw × d))	RCR *	RCR
Mean	<<0.00001	<<0.0001	0.0185	0.0185	0.0185
Maximum	<<0.00001	<<0.0001	0.0646	0.0646	0.0646

All values rounded to three significant figures, but unrounded values used for calculation. * Considers infrequent use of this product justified above (not applied to the inhalation exposure estimate, since the concentration in air is negligible already under default assumptions).

**Table 7:** Mean pathway-specific and aggregate RCRs of the main assessment (in bold) and considering additional uses for toddlers and adults (in italics).

Population Group	Food *	Drinking Water	Textiles	Consumer Use	Aggregate RCR
**Infants**	**0.0276**	**0.0472**	**0.0672**	**Irrelevant**	**0.142**
**Toddlers**	**0.0296**	**0.0405**	**0.0672**	**Irrelevant**	**0.137**
Toddlers ***	*0.0296*	*0.0405*	*0.0672*	*0.0306*	*0.168*
**Other children**	**0.0195**	**0.0312**	**0.0441**	**Irrelevant**	**0.0948**
**Adolescents**	0.0231 **	**0.0198**	**0.0441**	**Irrelevant**	**0.0870**
**Adults**	**0.0157**	**0.0160**	**0.0294**	**0.0185**	**0.0796**
Adults ***	*0.0157*	*0.0160*	*0.0294*	*0.0332*	*0.113*
**Elderly**	**0.00882**	**0.0124**	**0.0294**	**Irrelevant**	**0.0506**
**Very elderly**	0.0227 **	**0.0115**	**0.0294**	**Irrelevant**	**0.0636**

* Canned food and soft drinks. ** Overestimated due to overestimated consumption of canned food items (see SMF, Section S1.2.2 for details). *** RCRs resulting from additional, unconfirmed consumer uses: bottled glue by toddlers and carpet glue by adults (see main text for details).

**Table 8:** Assumptions in the mean risk estimates.

Exposure Pathway	Assumptions Representing	
	Mean Estimates	More Conservative Estimates
Canned food and soft drinks	Mean consumption data	Several canned food items added up per population group
		Canned/jarred food items assumed to be all in cans
		All soft drinks assumed to be in cans
		Only data for consumers of a food item considered (with a small fraction of consumers for some food items)
Textiles	Mean SA/BW ratio	60% of the skin surface area assumed to be covered by textile
		‘Worst-case’ migration rate
		High elastane fraction in textiles assumed
		Daily use of such textiles assumed

**Table 9:** Summary of information required for the case study by data accessibility for pathways of exposure.

Exposure Pathway	Assumptions Representing	
	Concentration of the Substance	Additional Input Parameters
**Public data for**		
Food and drinking water	SML and MTCtap (EU legislation)	Comprehensive Database (EFSA)
Textiles	Additional data to calculate concentration in garments	SA/BW ratio (US EPA) and additional default data (BfR
Consumer products	Typically not available	ConsExpo Web tool/fact sheets (RIVM)
**Non-public data for**		
Food and drinking water	Data from migration studies	Not needed
Textiles	Concentrations in mixtures/textile fibres	Not needed
Consumer products	Concentrations in end-use products	Not needed

## Data Availability

The original contributions presented in this study are included in the article/Supplementary Materials. All sources are publicly available except for the substance-specific concentrations in FCM, textiles and consumer products.

## Supplementary Materials

The following supporting information can be downloaded at: https://www.mdpi.com/article/10.3390/toxics14020165/s1, Table S1: Mean food and drinking water consumption data (intake in g/(kg bw × d) per country and population group; Table S2: Number of canned food items per population group and country; Figure S1: Comparison of mean food consumption derived in this report with EFSA data for total mean food consumption; Table S3: Comparative evaluation for adults based on EFSA default values; Table S4: SA/BW values and derived values for use in the exposure assessment; Table S5: ConsExpo input and results: Main assessment—Joint sealant (confirmed use); Table S6: ConsExpo input and results: Product used in higher amounts—Carpet glue (unconfirmed use); Table S7: ConsExpo input and results: Product used more frequently and potentially by children—Bottled glue (unconfirmed use); Table S8: Maximum and mean oral exposure and risk: Food and drinking water; Table S9: Alternative approach for nappies; Table S10: Mean and maximum RCRs for the three products assessed; Table S11: Fractions of pathway-specific RCRs of the aggregate RCRs by population group; Table S12: Evaluation of authorized substances in plastic FCM.

## Disclaimer

The information, statements, findings, opinions and data (together “INFORMATION”) contained in this publication are solely those of the individual author(s) and contributor(s)/consultant(s) and corresponds to the present state of their knowledge. Any user of the INFORMATION in this publication or the product(s) described therein is responsible for determining the suitability for its particular application. TO THE MAXIMUM EXTENT PERMITTED BY LAW, NO EXPRESS OR IMPLIED WARRANTY IS MADE OF THE MERCHANTABILITY, SUITABILITY, FITNESS FOR A PARTICULAR PURPOSE OR OTHERWISE OF ANY INFORMATION OR PRODUCT IN THIS PUBLICATION, NEITHER BY THE AUTHOR(S) AND CONTRIBUTOR(S)/CONSULTANTS(S) NOR BY CLARIANT LTD OR ANY OF ITS AFFILIATED GROUP COMPANIES. ANY RESPONSIBILITY FOR ANY INJURY TO PEOPLE OR PROPERTY RESULTING FROM ANY INFORMATION OR PRODUCT REFERRED TO IN THIS PUBLICATION IS REJECTED.

## Abbreviations

The following abbreviations are used in this manuscript:

ADI	Acceptable Daily Intake
ANSES	Agence Nationale de Sécurité Sanitaire de L’alimentation, de L’environnement et du Travail (French Agency for Food, Environmental and Occupational Health & Safety)
BfR	Bundesinstitut für Risikobewertung (German Federal Institute for Risk Assessment)
CAS	Chemical Abstract Service
CHESAR	Chemical Safety Assessment and Reporting
CLP	Classification, Labelling and Packaging
CMR	Carcinogenic, Mutagenic and Reprotoxic
CSS	Chemicals Strategy on Sustainability
DIY	Do-It-Yourself
DNEL	Derived No-Effect Level
EC	European Commission
ECETOC	European Centre for Toxicology and Ecotoxicology of Chemicals
ECHA	European Chemicals Agency
EFH	Exposure Factors Handbook
EFSA	European Food Safety Authority
EMA	European Medicines Agency
EU	European Union
EUPL	European Union Positive List
FCM	Food Contact Materials
GLP	Good Laboratory Practice
Log Kow	Logarithm of the Partition Coefficient between n-octanol and Water
MS	Member State (of the EU)
MTCtap	Maximum Tolerable Concentration at the Tap
OECD	Organization for Economic Co-operation and Development
OSOA	One Substance, One Assessment
RAC	Committee for Risk Assessment (of ECHA)
RCR	Risk Characterization Ratio
REACH	Registration, Evaluation and Authorisation of Chemicals
RIVM	Rijksinstituut voor Volksgezondheid en Milieu (Dutch National Institute for Public Health and the Environment)
SA/BW	Surface Area/Body Weight
SCF	Scientific Committee on Food
SMF	Supplementary Materials File
SML	Specific Migration Limit
STOT-RE	Specific Target Organ Toxicity following Repeated Exposure
TDI	Tolerable Daily Intake
TG	Test Guideline
TRA	Targeted Risk Assessment
US EPA	United States Environmental Protection Agency
