# Supplementary material for: Aggregate Consumer Exposure and Risk Assessment in the EU—A Case Study
Source: Toxics. 2026 Feb 11;14(2):165. doi: 10.3390/toxics14020165 (PMC12945028; doi:10.3390/toxics14020165)
Supplement: Supplementary file 1 [file toxics-14-00165-s001.zip › toxics-4088968-supplementary.pdf]

Supplementary Information File (SIF)

Aggregate Consumer Exposure and Risk Assessment in the EU – A Case Study

Jan Oltmanns, Christoph Scheibelein, Fabian A. Grimm

This SIF provides detailed information with respect to materials and methods, including justification of specific input values used in the exposure assessment (section SS1). Section SS2 provides more detailed results than is included in the main text. Section SS3 describes the additional evaluations performed to inform the discussion.

Contents

S1 Materials and Methods: Detailed information 2

S1.1 Food and drinking water limit values 2

S1.2 Data relevant for oral exposure from food and drinking water 2

S1.2.1 Hostanox® O 3 concentration in canned food items based on migration studies 2

S1.2.2 Food consumption data 2

S1.3 Data relevant for dermal exposure from textiles 10

S1.4 Data relevant for inhalation and dermal exposure from the use of sealants and other potential consumer products 12

S2 Results: Detailed information 15

S2.1 Exposure and risk from food and drinking water 15

S2.2 Exposure from textiles: nappies 17

S2.3 Exposure from the use of sealants and other potential consumer products 18

S2.4 Aggregate exposure and risk from all sources 18

S3 Additional evaluations 20

S4 References 22

S1 MATERIALS AND METHODS: DETAILED INFORMATION

S1.1 Food and drinking water limit values

The following limit values reported in the main text were derived from the Tolerable Daily Intake (TDI) of 0.1 mg/(kg bw x d) [1]:

- Food: A Specific Migration Limit (SML) of 6 mg/kg food was derived under the assumption of (a) a daily consumption of 1 kg of food and (b) a body weight of 60 kg [2]. This SML is included in Regulation (EU) No 10/2011.
- Drinking water: According to Commission Implementing Decision (EU) 2024/365 a Maximum Tolerable Concentration at the tap (MTCtap) is derived by dividing the SML by 20 L/kg. In line with these provisions an MTCtap for Hostanox® O 3 of 0.3 mg/L is included in the Commission Implementing Decision (EU) 2024/367, the positive list of starting substances, compositions and constituents authorized for use in the manufacture of drinking water contact materials. According to this legal act, MTCtap '*means the maximum permitted concentration of a substance transferred from a specific material into water intended for human consumption*'.

S1.2 Data relevant for oral exposure from food and drinking water

S1.2.1 Hostanox® O 3 concentration in canned food items based on migration studies

The migration of the substance into food simulants was investigated in a migration study in 2005 [3] for submission to the European Food Safety Authority. The preliminary migration study showed a Hostanox® O 3 concentration of 0.354 mg/kg food simulant (95% ethanol) over a period of 10 days at 40°C (mean of two duplicates: 0.372 and 0.336 mg/kg) from the material (low-density polyethylene (LDPE) plates containing 0.11% Hostanox® O 3 as measured in the study) at a surface-to-volume ratio of 6 dm^2/kg.

The full-scale migration study investigated migration from LDPE plates containing 0.11% Hostanox® O 3 (measured) into four simulants: (a) ethanol 95%, (b) ethanol 10%, (c) acetic acid 3% and (d) iso-octane (migration over 10 days at 40°C for all simulants except iso-octane (over 2 days at 20°C). The limits of quantification were 0.46 mg/kg (simulants (a)-(c)) and 0.15 mg/kg (simulant (d)). For each of the four simulants, migration was tested in triplicate. No migration of Hostanox® O 3 in simulants (a)-(c) was detectable (i.e. <0.46 mg/kg food). A mean migration of 0.20 mg/kg food was measured for simulant (d) with individual values in the triplicate experiments of 0.18, 0.19 and 0.23 mg/kg.

Based on these data, a mean concentration in food of 0.2 mg/kg and a maximum concentration in food of 0.5 mg/kg is used in the exposure assessment. The maximum is based on the limit of quantification for simulants (a)-(c) rounded to one significant figure.

S1.2.2 Food consumption data

Data extraction

Consumption data were extracted from EFSA's Comprehensive European Food Consumption Database [4], referred to below as 'Comprehensive Database'. The Comprehensive Database can be accessed on the EFSA website (https://www.efsa.europa.eu/en/data-report/food-consumption-data) and data can be downloaded in spreadsheet format. Consumption data were extracted in early 2024. While it is noted that datasets from Poland and Croatia were updated in December 2024, this database update is expected to have no significant impact on the assessment presented here.

The Comprehensive Database allows extraction of food consumption data at different levels of detail for specific food items (including drinking water), ranging from a very broad ('exposure hierarchy level 1' (L1) in EFSA's terminology) to rather detailed ('exposure hierarchy level 7' (L7)) descriptions. For example,

- 'vegetables and vegetable products' (L1) include for example
  - 'bulb vegetables' (L2),

- ‘fruiting vegetables’ (L2) and
- ‘processed or preserved vegetables and similar’ (L2), which include for example
    - ‘dried vegetables’ (L3),
    - ‘salted vegetables’ (L3) and
    - ‘canned/jarred vegetables’ (L3), which include e.g. at the most detailed L7 items such as ‘sweet corn canned’ and ‘canned mushrooms’.

Similar differentiations exist for soft drinks (e.g. ‘soft drink, lemon flavour’ and ‘soft drink, mixed flavours’) and to a somewhat lesser extent for drinking water (see below). While it is possible to extract information at the most detailed level (L7), adding up such L7 consumption data would result in overestimates since subjects consuming e.g. ‘canned mushrooms’ or ‘soft drinks, lemon flavour’ on a given day are unlikely to consume on the same day all other products defined at L7. An extraction at L3 or L4 provides a meaningful approach for the purpose of this exposure and risk assessment (also see discussion below).

Based on the use of Hostanox® O 3, consumption data were extracted for the following food items[1]:

- Canned food: Consumption is estimated based on L4 entries, since L3 entries do not differentiate canned products from un-canned in some cases (fish, seafood and fruit), while they do for other (e.g. vegetables and meat): typically, consumption data for about 3-9 canned food items are available per population group (based on median values in Table S2), with substantial differences between population groups and countries. The entries with available consumption data were added up per country and population group, resulting in conservative estimates as discussed below.
- Soft drinks: one L3 entry (soft drinks) is relevant and no addition of different L3 entries needs to be performed. No differentiation of canned soft drinks *vs.* soft drinks in other packaging (e.g. glass or plasticized carton) is possible.
- Drinking water: two L3 entries (drinking water and unbottled water) are potentially relevant; if both are reported for a population group in a specific country, typically one of these two entries has the largest fraction of consumers (i.e. those who actually consume the item among all participants in the survey). For example, 98% of all participants may consume unbottled water and 2% may consume drinking water. In this case, the consumption data for the first entry was used.

Apart from the food/drinking water items covered, data extraction followed some general principles:

- Only data for EU member states were included in the evaluation and data for other states (e.g. Montenegro and Serbia) were excluded.
- For each country and population group, only data from the most recent survey were included using the filter within the Comprehensive Database web interface. As discussed below, data from old and new surveys are apparently nonetheless selected for reasons unknown to us.
- Data were extracted for consumers only, i.e. the statistical descriptors for consumption data relate only to those persons consuming the respective food item. This represents a conservative approach since consumption data for all subjects are always lower. For example, the mean consumption of ‘canned mushrooms’ by Austrian adults who actually consume this food item is about 58 g/day (N=9), while it is 0.24 g/day among all 2169 participants in the survey due to a ‘dilution’ effect. The fact that some food items are consumed by a small fraction of the population is neglected (but see discussion below).
- The EFSA database includes consumption data in two different units: (a) consumption in g food per day and (b) consumption in g food/kg bw per day. The latter values were extracted since they allow calculation of oral exposure without assumptions on the body weight of specific population groups. This approach provides less uncertain estimates especially for the population groups of infants, toddlers and other children for which a single ‘average’ body weight neglects the fact that body weight rapidly increases with age.

[1] It is outside the scope of this document to presents EFSA’s food categorization system.

- Only population groups that have data from more than five countries were included in the extraction. Consequently, data for vegetarians and lactating women were excluded. Nonetheless, data are available for the most relevant age groups ranging from infants to very elderly persons (see Table S1) as described in EFSA [5][2].
- Data per country and population group were kept separate, i.e. no aggregation across age groups was performed, which is also not recommended in EFSA [5].

Note that not all food items are reported in all countries for all population groups (see Tables S1 and S2).

Evaluation of food consumption data

The extracted consumption data were evaluated considering that aggregate exposure is assessed by (a) combining oral exposure from the use in FCM and in drinking water and (b) combining total exposure from several sources, i.e. food, drinking water, textiles and consumer uses (where relevant). Since a combination of upper percentiles for several food items and/or pathways of exposure would result in gross overestimates, mean consumption data are used in the evaluation. For example, adding up the 95th percentile consumption data for all canned food items is unrealistic given that those who consume large amounts of one canned food item are unlikely to consume such large amounts of all other canned food items (as well as all soft drink items and drinking water) on the same day. It must also be noted that the upper percentiles reported are not robust for several food items (these are flagged in the extractions downloaded from the EFSA website; also see EFSA [5]) due to the small number of people who actually consume some of the food items covered. Also, for this reason an evaluation based on means provides more robust information.

The evaluation approach therefore followed the following principles:

- For each population group and country, the
  - mean consumption of all canned food items (sum of several items)
  - mean consumption of soft drinks
  - mean consumption of tap water

  is added up, resulting in a combined food/drinking water consumption.
- For each population group, the maximum combined food/drinking water consumption in any EU member state is evident (Table S1) and the values for (a) food consumption (canned food and soft drinks) and drinking water consumption associated with this maximum estimate across the EU is taken as an input for the exposure estimate. This approach ensures adequate consideration of potential combined oral exposure without adding up for a particular population group e.g. high oral intake via drinking water from one country and high oral intake via canned food from another country.

[2] Data for the population group 'pregnant women' were extracted as well, but not considered further, since these are lower than those for adults.

Table S1 **Mean food and drinking water consumption data (intake in g/(kg bw x d) per country and population group**

Propulation	Infants				Toddlers				Other children				Adolescents				Adults				Elderly				Very elderly			
**Country**	**Can**	**DW**	**Soft**	**∑**	**Can**	**DW**	**Soft**	**∑**	**Can**	**DW**	**Soft**	**∑**	**Can**	**DW**	**Soft**	**∑**	**Can**	**DW**	**Soft**	**∑**	**Can**	**DW**	**Soft**	**∑**	**Can**	**DW**	**Soft**	**∑**
Austria									2.35	17	5.55	25.0	4.65	20.0	6.75	31.4	6.01	19.7	5.22	30.9		8.6	3.55	12.2	0.08	13.9	2.84	16.8
Belgium					5.32	4.9	13.5	23.7	12.9	17	10.5	40.2	6.10	9.1	7.42	22.7	6.70	7.0	5.70	19.4	3.47	5.4	2.95	11.8	3.43	4.7	2.97	11.1
Bulgaria	**7.38**	**53.5**	**16.1**	**77.0**	5.95	30.2	8.53	44.7	5.12	28	9.47	42.8																
Croatia																	0.55	15.2	3.44	19.2								
Cyprus	0.43	19.3		19.7	2.12	21.1	0.65	23.9	1.26	16	3.46	20.6	2.06	9.7	3.05	14.8	2.45	7.5	2.68	12.6	2.71	8.2	2.28	13.2				
Czechia									7.08	19	10.6	36.6	3.84	12.9	8.19	24.9	3.09	11.7	4.89	19.7								
Denmark	1.47	33.4	3.02	37.9	1.57	30.2	3.72	35.4	0.982	22	6.01	28.8	0.589	14.3	4.73	19.6	0.46	13.0	3.22	16.7	0.41	10.5	1.79	12.7	0.28	9.1	0.74	10.1
Estonia	6.64	12.3	5.82	24.8	6.28	23.0	7.57	36.8	5.13	16	6.08	27.0	5.24	22.4	9.02	36.6	2.75	9.0	2.50	14.3	2.60	8.3	1.33	12.2				
Finland	1.04	30.1		31.2	3.71	44.8	2.25	50.8	1.56	18	4.60	24.3	0.987	11.8	3.47	16.2	1.92	14.0	3.19	19.1	2.68	9.0	2.52	14.2				
France	1.99	10.6	2.08	14.7	13.07	19.0	8.14	40.2	10.7	18	6.35	35.1	6.82	10.1	4.19	21.1	5.72	9.5	3.24	18.5	5.20	7.4	2.52	15.1	5.29	7.5	1.32	14.1
Germany	2.05	20.2	8.17	30.4	3.02	16.6	15.50	35.1	6.84	18	21.1	46.2	**10.6**	**12.0**	**14.2**	**36.8**	8.81	7.7	5.69	22.2	8.30	6.1	3.45	17.9	4.93	5.5	3.83	14.2
Greece									2.56	14	7.33	24.3	1.74	5.1	3.56	10.4	1.23	6.8	2.99	11.0	1.41	4.8	3.20	9.39				
Hungary					**9.10**	**45.9**	**16.1**	**71.1**	**6.94**	**35.4**	**9.60**	**52.0**	4.45	20.3	6.52	31.2	5.40	15.3	4.49	25.2	**3.61**	**14.0**	**3.89**	**21.5**	1.28		2.39	3.67
Ireland																	1.55	7.4	2.86	11.9	1.14	5.7	2.05	8.89	2.25	5.7	1.51	9.48
Italy	0.02	25.7		25.7	1.94	26.7	6.63	35.3	5.65	26	6.59	38.1	3.16	17.1	4.57	24.8	2.83	10.5	2.72	16.0	1.81	9.7	1.46	13.0	0.72	5.1	1.57	7.35
Latvia	8.58	15.9	13.3	37.9	4.45	15.5	23.40	43.3	6.06	17	5.58	28.2	4.35	8.2	3.43	15.9	3.70	3.9	3.05	10.6	3.39	4.6	2.75	10.7	0.32	3.9		4.20
Netherlands					20.92	27.0	17.92	65.9	13.5	20	12.6	46.1	6.92	12.1	8.61	27.6	6.46	10.4	5.30	22.1	6.03	7.1	2.98	16.1	**14.6**	**13.0**	**4.72**	**32.3**
Portugal	1.54	19.1	18.0	38.7	5.12	17.9	8.01	31.0	4.37	13	7.86	25.5	2.11	10.5	6.10	18.7	2.17	10.0	3.76	16.0	2.02	9.7	2.70	14.4	1.32	10.0	2.62	13.9
Romania													1.54	14.8	5.28	21.6	4.91	9.1	4.18	18.2	2.49	9.1	2.72	14.3	0.21	3.1	1.11	4.39
Slovenia	4.13	19.5	1.79	25.4	6.92	25.1	7.81	39.9					5.33	13.5	4.24	23.0	4.57	11.6	2.81	19.0	4.20	8.8	2.97	16.0				
Spain		7.56		7.56	6.39	17.2	6.03	29.7	4.15	14	5.93	23.9	3.12	9.3	3.95	16.4	5.33	7.8	3.11	16.3	3.42	6.8	1.69	11.9				
Sweden									4.32	7	9.52	20.7	3.68	9.6	6.19	19.5	**4.22**	**18.1**	**9.12**	**31.4**	0.90	6.9	1.78	9.56	1.06	6.3	1.84	9.25
**Evaluation**																												
**Maximum**				**77.0**				**71.1**				**52.0**				**36.8**				**31.4**				**21.5**				**32.3**
Can				7.38				9.10				6.94				10.6				4.22				3.61				14.6
Soft				16.1				16.1				9.60				14.2				9.12				3.89				4.72
DW				53.5				45.9				35.4				12.0				18.1				14.0				13.0
Country*	Bulgaria				Hungary				Hungary				Germany				Sweden				Hungary				Netherlands			

Can: Canned food; DW: Drinking water; Soft: Soft drinks. * Country showing the maximum combined consumption (∑) for each age group.
No emphasis was placed on the number of significant figures for this presentation exact for values driving the final consumption estimate, i.e. the maximum combined consumption in each population group, which were rounded to three significant figures. All calculations are based on unrounded values as extracted from the Comprehensive Database.
Source: data calculated from original data in EFSA's Comprehensive Database as described above.

Table S2 **Number of canned food items per population group and country**

	Infants	Toddlers	Other children	Adolescents	Adults	Elderly	Very elderly
Austria			4	9	11		1
Belgium		4	13	12	13	6	7
Bulgaria	**3**	3	4				
Croatia					1		
Cyprus	1	4	4	5	6	6	
Czechia			7	5	7		
Denmark	6	6	6	6	6	6	5
Estonia	5	8	9	14	9	9	
Finland	1	4	3	3	6	6	
France	1	9	12	13	13	12	11
Germany	3	7	14	**18**	14	13	9
Greece			4	4	4	2	
Hungary		**7**	**7**	8	10	**8**	2
Ireland					5	4	4
Italy	1	4	6	6	7	5	4
Latvia	4	6	7	8	9	8	2
Netherlands		14	13	13	14	11	**21**
Portugal	1	4	4	4	5	5	4
Romania				5	9	6	1
Slovenia	3	6		7	5	7	
Spain		5	6	6	9	6	
Sweden			9	8	**12**	4	4
**Evaluation**							
Arithmetic mean	2.6	6.1	7.3	8.1	8.3	6.9	5.8
Median	3.0	6.0	6.5	7.0	9.0	6.0	4.0
Range	1-6	3-14	3-14	3-18	1-14	2-13	1-21

Country-population group combinations used for the exposure estimate (see Table S1) are highlighted.

Comparison of derived food consumption data with other data

To assess the plausibility and the degree of conservatism, the calculated mean consumption of canned food and soft drinks derived as the final input for the exposure estimate given in g food/(kg bw x d) (Table S1) was compared with the total mean food consumption (solid & liquid) reported in EFSA [6] for the population groups covered by this assessment[3]. This source provides three different values for infants and the mean of these three values was used for this comparison. The following plot shows the data for this comparison.

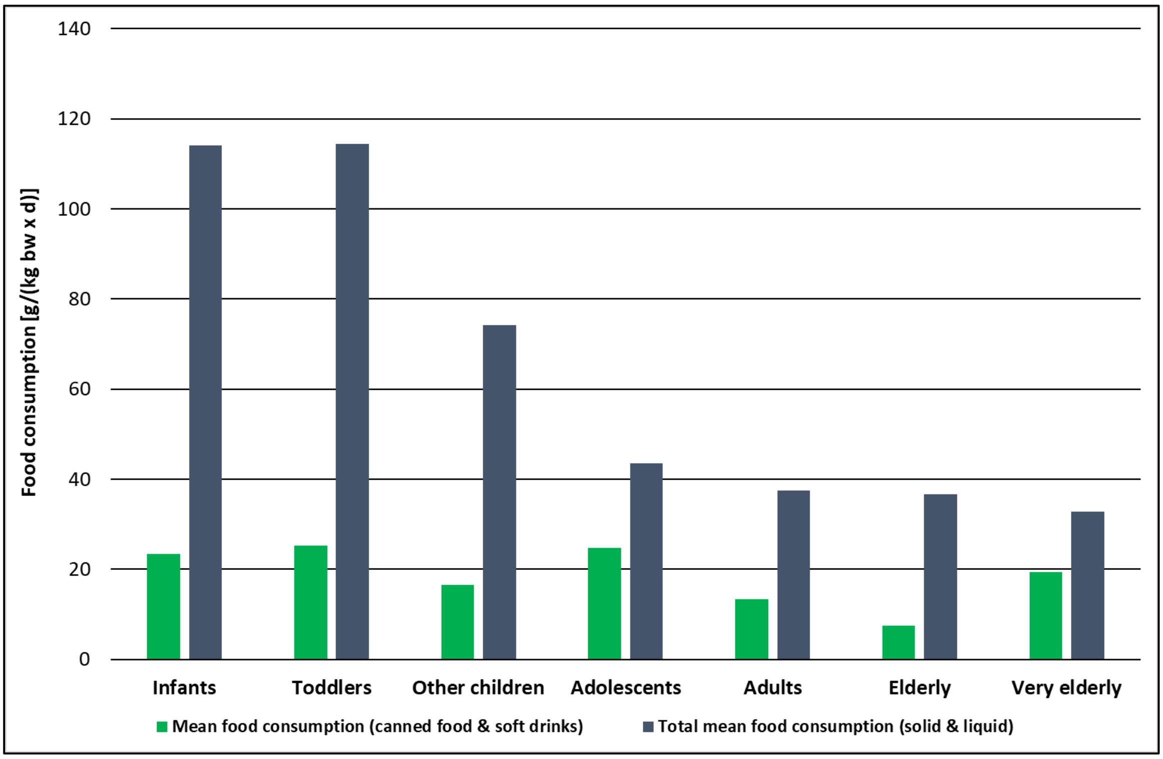

Figure S1 **Comparison of mean food consumption derived in this report with EFSA data for total mean food consumption**

This comparison illustrates the following points:

- Total mean food consumption adjusted for body weight according to the EFSA data decreases with increasing age, which is explained in the EFSA report: '*Infants and toddlers have higher food intake than older children, adolescents or adults expressed on a body weight basis due to their higher energy requirement during rapid growth*' [6].
- The calculated mean consumption of canned food and soft drinks derived in this report generally follows this trend with two notable exceptions (see Table S1):
  - Adolescents have a higher mean consumption of canned food and soft drinks than younger children (except toddlers).
  - Very elderly people have a higher mean consumption of canned food and soft drinks than adults and the elderly.

  In both cases, the underlying data from Germany (adolescents) and the Netherlands (very elderly) show exceptionally high numbers of 18 (adolescents) and 21 (Netherlands) canned food items (see Table S2). A more detailed analysis of the consumption data extracted shows double counting of some canned food items for these datasets, which results in overestimates[4]. The

[3] The EFSA data are assumed not to cover drinking water intake, since this is covered separately in the document.

[4] More specifically, data for the same L4 food item (e.g. 'canned or jarred fruit') from different surveys is added up by our evaluation approach, even though only the most recent surveys were selected (see above). Apparently, this only applies to identical surveys repeated over time, but not to different surveys. For example, the German data for adolescents include

data in Table S1 demonstrate that such high consumption values are not noted in any other country. Since the estimated oral exposure to Hostanox® O 3 is low (see main text) no further refinement was performed, but the data for adolescents and very elderly people should not be used for risk assessment purposes.

- Due to these overestimates the amounts of canned food and soft drinks consumed by adolescents and very elderly amount to almost 60% of the total solid and liquid food consumption, again demonstrating the gross overestimate. For the three youngest age groups, this fraction is slightly above 20%, while for adults it is about 35%.

The more detailed data in Table S1 indicate that the consumption of soft drinks exceeds the one of canned food items except for the very elderly due to the overestimate of canned food items discussed above.

The food consumption data derived are considered conservative for the following reasons:

- Canned food items
  - Several of the L4 entries evaluated cover canned or jarred food items, resulting in an overestimate with respect to canned food.
  - Consumption of all canned food items is added up, while in fact persons consuming one canned food item on a particular day may not consume other canned food items. For example, the evaluation approach assumes that Hungarian toddlers (which show the highest consumption of canned food (see Table S1) and were therefore used for the exposure assessment) consume on the same day (a) canned or jarred fruit, (b) canned or jarred chickpea, (c) canned or jarred peas, (d) canned-tinned meat, (e) canned/ jarred vegetables, (f) sweet corn canned and (g) canned/jarred fish (N=7; see Table S1).
  - The fraction of consumers is small for several canned food items due to the evaluation at L4 that was necessary to cover all canned food items. For the example of Hungarian toddlers (see above), five of the seven canned food items are consumed by only 3% of survey participants or less (maximum: 37.2% for canned/jarred vegetables).
- Soft drinks
  - The L3 entry ‘soft drinks’ does not differentiate between soft drinks in cans and in other packaging. Such a differentiation is also not available at the more detailed levels L4-L7. The evaluation therefore provides an upper end mean estimate with respect to canned soft drinks.
  - The fraction of consumers is comparatively low for the younger age groups. With respect to the data used for the exposure assessment, it is only 0.7% for Bulgarian infants, 7.7% for Hungarian toddlers and 21% for Hungarian ‘other children’.
  - For adolescents, the German data that form the basis of the exposure assessment involve double counting since data from two surveys are added up (see discussion above). As noted for canned food items, such high values are not observed in other countries, but input data were not refined since oral exposure is low even in this case.

With respect to drinking water, the approach applied does not involve any of the conservative elements discussed above. In contrast, the evaluation may somewhat underestimate exposure since it is limited to drinking water (generally reflecting unbottled/tap water) but may not consider beverages prepared from drinking water (e.g. tea and coffee). The adequacy of the values derived for exposure and risk assessment can be checked against default values derived by EFSA for adults. The estimated mean drinking water consumption of 18.1 g/(kg bw x d) shown in Table S1 results in a drinking water intake of 1.27 L/d (70 kg bw assumed; see [6]). Together with a soft drink consumption of 0.639

consumption data from the ‘Eating Study as a KiGGS Module’ started in 2006 as well as from the ‘National Nutrition Survey II’ started in 2007. A total of 11 distinct canned food items is covered with data from both surveys for 7 of these (N=18 in total). The Dutch data for the very elderly involve 12 distinct canned food items with data from two surveys for 9 of these (N=21 in total).

L/d calculated for the same group under identical assumptions, the total intake of drinking water and soft drinks is 1.91 L/d. The derived value is just below the EFSA default value of 2 L/d '*for chronic daily total liquid intake (i.e. milk, tap water, other beverages) (…) recommended for European adults, including the elderly*' [6]. Since the latter includes other beverages than drinking water and soft drinks (e.g. milk), the derived value is considered conservative for adults. For the elderly (also covered by the EFSA default value), similar calculations result in a consumption of soft drinks and drinking water of 1.25 L/d, which is considered an adequate estimate. EFSA [6] does not provide default values for liquid intake for other population groups.

We conducted additional analyses based on default consumption data of solid food and liquid intake established by EFSA for adults[5]. For this comparison, the following EFSA default values for adults are used:

- Default solid food intake: 1 kg/d is assumed for an adult with a body weight of 60 kg [2]. These assumptions are also used to derive SMLs for FCM. While this value is not necessarily limited to solid food, it is discussed as a default for solid food by EFSA's Scientific Committee [6], although not recommended (see discussion below). This food intake corresponds to a consumption normalized to body weight of 16.7 g/(kg bw x d).
- Default liquid intake: EFSA's Scientific Committee '*recommends using 2 L as a default value for chronic daily total liquid intake (i.e. milk, tap water, other beverages) by adults to be used in risk assessments*' [6]. The same source identifies 70 kg as the default body weight for European adults, resulting in a default liquid consumption normalized to body weight of 28.6 g/(kg bw x d).

Table S3 compares the default consumption data for solid food and liquids with the ones used in this study.

Table S3 Comparative evaluation for adults based on EFSA default values.

	Food consumption (g/(kg bw x d)		
	**Based on EFSA default values**	**Applied in this study**	**Fraction**
Solid food	16.7	4.22*	25%
Liquids	28.6	27.2**	95%

* Canned food (see Table 3 in the main text). ** Soft drinks and drinking water combined (see Table 3 in the main text).

This comparison demonstrates that the approach applied in this study is unlikely to underestimate exposure when compared with EFSA default values. First, the consumption of canned food items assumed in this study accounts for 25% of the EFSA default value for all solid foods. In fact, under the assumption of a body weight of 60 kg (see EFSA default value above), the value of 4.22 g/(kg bw x d) corresponds to an intake of 253 g canned food per day. Second, the consumption of soft drinks and drinking water accounts for 95% of the EFSA default value for all liquids. This is considered to represent a conservative approach. With the default body weight of 70 kg (applied for liquid intakes as discussed above), the value derived in this study of 27.2 g/(kg bw x d) corresponds to an intake of 1.90 L per day, of which 1.27 L/d is assumed to be drinking water based on the data presented in Table 3. It must be stressed that the consumption data used in this study represent mean values.

While a default food consumption value is used by EFSA's Panel on Food Contact Materials, Enzymes, Flavourings and Processing Aids [2] for setting SMLs and used here for solid food intake, EFSA's Scientific Committee found it impossible to derive a default value for solid food intake, but rather stated: *'The Scientific Committee (…) recommends that the EFSA Comprehensive Database is regularly*

[5] This evaluation differs from the one presented above by using default intakes and default body weights to calculate consumption for solids and liquids normalized to body weight for adults only, whereas the EFSA data used above are already given as consumption normalized to body weight for all population groups.

consulted to check the relevance of the default values that are used' [6]. The use of the Comprehensive Database for food consumption data as applied in this study is fully in line with these provisions and, in fact, is considered superior to the use of the default intake values shown in Table S3. Specifically, using consumption data normalized to body weight as provided in the Comprehensive Database does not require assumptions on the body weight that differ between and even within risk assessment agencies as evidenced e.g. by the default body weights of 60 and 70 kg above. More generally, different assumptions on body weights and other input data in various European regulatory areas were recently highlighted [7] and the consistent use of the Comprehensive Database for dietary exposure assessments was recommended [7,8].

Taken together, the analyses presented above indicate that the approach used in this study is unlikely to underestimate mean consumption of canned food, soft drinks and drinking water.

S1.3 Data relevant for dermal exposure from textiles

The German Federal Institute for Risk Assessment (BfR) provides default values for the assessment of dermal exposure to additives in textiles [9]. These following default values are intended to provide worst-case estimates in the absence of specific data:

- Skin surface area in contact with the textile: 1 m²
- Specific weight of the textile: 100 g/m²
- Migration rates, depending on substance type
  - Textile dyes: 0.5%
  - Hydrophobic textile additives: 0.1%
  - Hydrophilic textile additives: 2%
- Body weight: 60 kg

While this information forms the basis of the exposure assessment, it is not used as such for the following reasons:

- The default values only relate to adults while the exposure assessment covers additional population groups. The BfR approach does not specifically discuss such an exposure assessment for children but only mentions that e.g. the body weight may need adaptation, if exposure of children is being assessed.
- The skin surface area and the body weight are not independent parameters but are related. Furthermore, the skin surface area of children – when normalized to body weight – is substantially higher than for adults. In fact, the surface area/body weight (SA/BW) ratio decreases considerably with age [10,11].

Consequently, data on the skin surface area in contact with textiles (and the resulting amounts of textile) in contact with the skin are first derived followed by a consideration of migration rates.

Skin surface areas in contact with textiles

Based on the considerations above, the following mean SA/BW values expressed in m²/kg bw were taken from the US EPA's Exposure Factors Handbook [10], which were derived from earlier work by others [11]:

- infants/toddlers (0-2 years of age): 0.064 m²/kg bw,
- children (2.1-17.9 years of age): 0.042 m²/kg bw and
- adults (18 years or older): 0.028 m²/kg bw.

Median values are identical or very similar to these mean values. These values reflect the total skin surface area for females and males combined [10], referred to as the total SA/BW below.

The default values in the BfR approach assumed a skin surface area in contact with the skin of 0.0167 m²/kg bw for adults (1 m² divided by 60 kg as shown above). This corresponds to 60% of the total SA/BW value of 0.028 m²/kg bw taken from US EPA [10]. In other words, the BfR approach implies that

60% of the total skin surface area is covered by the textile. Based on the recommended values for the mean skin surface area of body parts in US EPA's Exposure Factors Handbook, this fraction of 60% approximately corresponds to the skin surface of the entire trunk, one half of the arms plus one half of the legs[6]. The assumption of a skin coverage of 60% is considered to represent a conservative estimate for most clothing, such as trousers/leggings (surface of legs accounting for <35% of the total skin surface for all age groups) and bathing suits (trunk accounting for <42 of the total skin surface for all population groups). While it is recognized that clothing containing elastane may cover larger skin surface areas (i.e. entire trunk, entire legs and entire arms, e.g. full-body gym suits), the assumption of 60% of the total skin surface being covered by the textile must be considered together with the high elastance fraction assumed (30%), which both combined taken as a conservative input. We therefore retain the 60% assumption resulting from the BfR default assumption.

The SA/BW in contact with textiles ('contact SA/BW' below) is calculated by multiplying the total SA/BW value for the different age groups by the fraction of 60%. The contact SA/BW derived by this approach results in a value for adults (0.0167 m²/kg bw) that is identical to the one implicit in the BfR approach (see above), but adequately covers the higher surface area per kg body weight in children and adolescents (see Table S4).

With the default specific weight of textiles of 100 g/m² [9], the amount of textile in contact with the skin can be calculated, again normalized to body weight (see Table S4). The resulting value for adults (1.67 g textile/kg body weight) results in a total amount in contact with the skin of 100 g when the BfR default body weight of 60 kg is used. This value appears rather low, a finding that is due to the comparatively low default specific weight used in the BfR approach. While BfR [9] does not discuss this value or its origin, it may be assumed that this low value aims at reflecting the innermost layer of the textile that is in direct contact with the skin. While this assumption cannot be verified, we refrain from using a higher specific surface area, since (a) the BfR considers its approach generally to be worst-case, (b) we assume a relatively high elastane fraction in the fabric (see section 2.4.2 in the main article) and (c) the fraction of the skin surface area covered by the textile of 60% is considered conservative as well. Table S4 summarizes the data derived based on the discussion above.

Table S4 SA/BW values and derived values for use in the exposure assessment

Population group	Total SA/BW (m²/kg bw)	Contact SA/BW (m²/kg bw)*	Specific textile weight (g/m²)	Amount of textile (g/kg bw)
Infants	0.064	0.0381	100	3.81
Toddlers	0.064	0.0381	100	3.81
Other children	0.042	0.0250	100	2.50
Adolescents	0.042	0.0250	100	2.50
Adults	0.028	0.0167	100	1.67
Elderly	0.028	0.0167	100	1.67
Very elderly	0.028	0.0167	100	1.67

* Skin surface area in contact with textiles.

The amount of textile (normalized to body weight) in the last column of the table is multiplied by the concentration of Hostanox® O 3 as derived in the main article, which results in an amount of Hostanox® O 3 present in the textile that is in contact with the skin. Again, this figure is expressed in units normalized to body weight. Not all of the substance present in the textile will be available for exposure, but only the fraction that migrates from the textile. This fraction is covered by the migration rate discussed in the main article.

[6] The fractions of these body parts of the total skin surface area add up to 53-54% for children up to an age of 2 years, 61-63% up to an age of 20 years and 64% (males) and 58% (females) for subjects 21 years of age or older.

S1.4 Data relevant for inhalation and dermal exposure from the use of sealants and other potential consumer products

Table S5 summarizes the input and output data of ConsExpo modelling for the main assessment that considers the confirmed use of the substance in joint sealants. Tables S6 and S7 provide input and output data for the additional products assessed (unconfirmed uses).

All input data other than the Hostanox® O 3 concentration are based on the ConsExpo fact sheet for DIY products [12] and on the ConsExpo general fact sheet [13] for body weights. Note that ConsExpo fact sheets are available for several other consumer products, such as air fresheners, cleaning products and paint products, on the ConsExpo website (https://www.rivm.nl/en/consexpo/ fact-sheets).

The body weight for children (25th percentile: 12.4 kg) is discussed in section 2.4.4 of the main text. ConsExpo includes three different default body weights for adults that represent 25th percentiles: 68.8 kg (males and females combined), 77.2 kg (males) and 64.1 (females) [13]. We used a body weight of 65 kg for the following reasons: First, this value represents the (rounded) female body weight in ConsExpo, therefore covering the use of products by females. The resulting dermal exposure estimate (0.0185 mg/kg bw at mean substance concentration) is only a marginally lower than the one resulting with the default ConsExpo female body weight of 64.1 kg (0.0187 mg/kg bw; details not shown). This minor difference is considered acceptable considering that use of the 25th percentile for the body weight involves a conservative element in the context of the aggregate exposure assessment. Second, using the adult (males and females combined) default body weight of ConsExpo (68.8 kg) would result in a lower dermal exposure estimate (0.0174 rather than 0.0185 mg/kg bw at mean substance concentration) that may not be sufficiently conservative. Finally, the value of 65 kg is the mean of the default adult body weights of 60 and 70 kg, respectively, used by EFSA as discussed in section 3.1 of the main text. It is therefore consistent with the EFSA approaches, although food intake values normalized to body weights are used in the assessment.

Conservative input values for the body weight are chosen since several other input values (e.g. on the contact rate and release duration) are somewhat uncertain based on the 'quality' rating in the ConsExpo fact sheet.

Table S5 ConsExpo input and results: main assessment – joint sealant (confirmed use)

Parameter	Input and Output parameters values		Unit
**Product**	**Joint sealant**		
	**Mean**	**Maximum**	
**Input**			
Molecular weight	795		g/mol
KOW	7		10Log
Weight fraction substance	0.08	0.28	%
Body weight	65 (adult)		kg
Frequency of use	3		per year
**Inhalation**			
Exposure model	Exposure to vapour - Evaporation		
Exposure duration	45		minute
Product in pure form	No		
Molecular weight matrix	3.00E+03		g/mol
Product amount	350		g
Room volume	10		m^3
Ventilation rate	2		per hour
Application temperature	20		°C
Vapor pressure	1.00E-06		Pa
Mass transfer coefficient	10		m/hr
Release area mode	Increasing		
Release area	0.025		m^2
Emission/application duration	30		minute
**Dermal**			
Exposure model	Direct contact - Constant rate		
Exposed area	30		cm^2
Contact rate	50		mg/min
Release duration	30		minute
Retention factor	1		
**Results**			
**Inhalation**			
Mean event concentration	3.14E-09	1.09E-08	mg/m^3
Peak concentration (TWA 15 min)	6.12E-09	2.13E-08	mg/m^3
**Mean concentration on day of exposure**	**9.80E-11**	**3.41E-10**	**mg/m^3**
Year average concentration	8.05E-13	2.80E-12	mg/m^3
**Dermal**			
Dermal load	0.04	0.14	mg/cm^2
External event dose	0.0185	0.0646	mg/kg bw
**External dose on day of exposure**	**0.0185**	**0.0646**	**mg/kg bw**
RIVM ConsExpo Web, version 1.1.1, 17-01-2023			

Table S6 **ConsExpo input and results: product used in higher amounts – carpet glue (unconfirmed use)**

Parameter	Input and Output parameters values		Unit
Product	Carpet glue		
	Mean	Maximum	
**Input**			
Molecular weight	795		g/mol
KOW	7		10Log
Weight fraction substance	0.08	0.28	%
Body weight	65 (adult)		kg
Frequency of use	1		per year
**Inhalation**			
Exposure model	Exposure to vapor - Evaporation		
Exposure duration	90		minute
Product in pure form	No		
Molecular weight matrix	3.00E+03		g/mol
Product amount	1.42E+04		g
Room volume	58		m^3
Ventilation rate	0.5		per hour
Application temperature	20		°C
Vapor pressure	1.00E-06		Pa
Mass transfer coefficient	10		m/hr
Release area mode	Constant		
Release area	4		m^2
Emission/application duration	90		minute
**Dermal**			
Exposure model	Direct contact - Constant rate		
Exposed area	113		cm^2
Contact rate	30		mg/min
Release duration	90		minute
Retention factor	1		
**Results**			
**Inhalation**			
Mean event concentration	3.03E-07	1.06E-06	mg/m^3
Peak concentration (TWA 15 min)	4.61E-07	1.60E-06	mg/m^3
**Mean concentration on day of exposure**	**1.90E-08**	**6.60E-08**	**mg/m^3**
Year average concentration	5.19E-11	1.81E-10	mg/m^3
**Dermal**			
Dermal load	0.0191	0.0669	mg/cm^2
External event dose	0.0332	0.116	mg/kg bw
**External dose on day of exposure**	**0.0332**	**0.116**	**mg/kg bw**
RIVM ConsExpo Web, version 1.1.1, 17-01-2023			

Table S7 **ConsExpo input and results: product used more frequently and potentially by children – bottled glue (unconfirmed use)**

Parameter	Input and Output parameters values		Unit
**Product**	**Bottled glue: universal/wood glue**		
	**Mean**	**Maximum**	
**Input**			
Molecular weight	795		g/mol
KOW	7		10Log
Weight fraction substance	0.08	0.28	%
Body weight	12.4 (child 2-3 year))		kg
Frequency of use	36		per year
**Inhalation**			
Exposure model	Exposure to vapor - Evaporation		
Exposure duration	240		minute
Product in pure form	No		
Molecular weight matrix	3.00E+03		g/mol
Product amount	10		g
Room volume	20		m^3
Ventilation rate	0.6		per hour
Application temperature	20		°C
Vapor pressure	1.00E-06		Pa
Mass transfer coefficient	10		m/hr
Release area mode	Increasing		
Release area	0.05		m^2
Emission/application duration	36		minute
**Dermal**			
Exposure model	Direct contact - Instant application		
Exposed area	15		cm^2
Product amount	0.080		g
Retention factor	1		
**Results**			
**Inhalation**			
Mean event concentration	2.2E-08	7.72E-08	mg/m^3
Peak concentration (TWA 15 min)	3.5E-08	1.2E-07	mg/m^3
**Mean concentration on day of exposure**	**3.7E-09**	**1.29E-08**	**mg/m^3**
Year average concentration	3.7E-10	1.27E-09	mg/m^3
**Dermal**			
Dermal load*	0.0043	0.0149	mg/cm^2
External event dose	0.0052	0.0181	mg/kg bw
**External dose on day of exposure**	**0.0052**	**0.0181**	**mg/kg bw**
RIVM ConsExpo Web, version 1.1.1, 17-01-2023			

* To be used with caution since ConsExpo does not adapt the exposed area (15 cm^2 assumed for adults) to the selected age group (children 2-3 years of age). The body weight of this age group, however, is correctly applied and the resulting external dose used in the exposure and risk assessment is correct: 80 mg product x 0.28% / 12.4 kg bw = 0.0191 mg/(kg bw x d).

S2 RESULTS: DETAILED INFORMATION

S2.1 Exposure and risk from food and drinking water

The following table does not add up exposure from all three categories since such an addition is inadequate for the maximum exposure estimates. The main article provides the combined mean oral exposure from all three categories and the resulting RCRs.

Table S8 Maximum and mean oral exposure and risk: food and drinking water

	Population group	Oral exposure (mg/(kg bw x d)		RCR	
		Maximum	Mean	Maximum	Mean
Canned food	Infants	0.00369	0.00148	0.0217	0.00868
	Toddlers	0.00455	0.00182	0.0268	0.0107
	Other children	0.00347	0.00139	0.0204	0.00816
	Adolescents	0.00530	0.00212	0.0312	0.0125
	Adults	0.00211	0.00084	0.0124	0.00496
	Elderly	0.00181	0.00072	0.0106	0.00425
	Very elderly	0.00730	0.00292	0.0429	0.0172
Soft drinks	Infants	0.00805	0.00322	0.0474	0.0189
	Toddlers	0.00805	0.00322	0.0474	0.019
	Other children	0.00480	0.00192	0.0282	0.011
	Adolescents	0.00451	0.00180	0.0265	0.011
	Adults	0.00456	0.00182	0.0268	0.0107
	Elderly	0.00195	0.000778	0.0114	0.00458
	Very elderly	0.00236	0.000944	0.0139	0.00555
Drinking water	Infants	0.0161	0.00803	0.0944	0.0472
	Toddlers	0.0138	0.00689	0.0810	0.0405
	Other children	0.0106	0.00531	0.0625	0.0312
	Adolescents	0.00672	0.00336	0.0395	0.0198
	Adults	0.00543	0.00272	0.0319	0.0160
	Elderly	0.00420	0.00210	0.0247	0.0124
	Very elderly	0.00390	0.00195	0.0229	0.0115

All values rounded to three significant figures, but unrounded values used for calculation.

S2.2 Exposure from textiles: nappies

As noted in the main article, dermal exposure from textile parts used as leg bands in nappies will be substantially lower than shown in the results section, since the skin surface area covered by such leg bands will be well below 60% of the total skin surface area as assumed in the main assessment.

An alternative approach was used to assess this suggestion. The French Agency for Food, Environmental and Occupational Health & Safety (ANSES) published a safety assessment for nappies which provides input data for an exposure assessment [14]. In line with the general approach described above, a mean and maximum estimate based on mean and maximum Hostanox® O 3 concentrations is provided using the 'refined scenario' (rather than 'worst case') input data from the ANSES report. These input data are provided in the ANSES report for six groups of children from 0 to 36 months of age, i.e. a higher granularity than is used for the EFSA population groups of infants and toddlers covering the same ages (see Table 2 in the main article). For each of these six groups, the weight of nappies, their frequency of use and the body weight is given in the ANSES report. Based on in-depth evaluations, these input data result in decreasing RCRs with increasing age (details not shown). Therefore, only input data and results for the two youngest age groups (largely conforming to the EFSA age group of infants) are shown in the following table. The comparative exposure assessment is based on the following input data and calculations:

- Weight of a single nappy and use frequency (number of nappies per day) taken from the ANSES report [14]; both values were multiplied resulting in the weight of nappies used per day.
- Of the total weight per day, leg bands only reflect a small fraction. Since no information on the weight of the leg bands in nappies could be located, a fraction of 2.5% is assumed. This fraction is based on the following assumptions for the youngest age group:
  - Diameter of one leg band (15 cm) and height of one leg band (2 cm), resulting in a surface area of 0.006 m² for both leg bands of a nappy.
  - Specific weight of leg bands (100 g/m²), resulting in a weight of the two leg bands of 0.6 g, which represents 2.5% of the total weight of 24 g (see Table S9). This fraction was applied to nappies for both age groups.

  The leg band weight for the youngest infants is therefore estimated to be (24 g/nappy x 7.98 nappies/day x 2.5% =) 4.79 g/d (see Table S9).
- This weight is multiplied by the Hostanox® O 3 concentration (mean and maximum) and the migration rate of 0.1% for hydrophobic textile additives [9] as discussed in the main text, resulting in the amount migrated (in mg/d).
- Finally, this migrated amount is divided by the body weight, resulting in the dermal exposure estimate (in mg/(kg bw x d)) and the corresponding RCRs. The ANSES report assumed body weights of 3.9 (0-5 months old infants) and 7 kg (6-12 months old infants) based on the representative French 'Nutri-bébé' survey. While a reference is provided within the report, the underlying data in Table 24 of the ANSES report only relate to children 0-1 year of age (5th percentile: 4.1 kg, median: 7.5 kg, range: 2.6-11.5 kg). The default values of 3.9 kg and 7 kg cannot be reproduced from these data and their degree of conservatism therefore remains uncertain. However, data from this French survey reported by Chouraqui*, et al.* [15] indicate that they represent lower percentiles of the distribution. In the light of these uncertainties, the following body weights were derived:
  - The arithmetic mean of the median body weights of boys and girls at 3 months and 9 months of age, respectively, was calculated from representative German data [16] for the two age groups considered[7].
  - The resulting values of 5.87 and 8.79 kg, respectively, agree well with

[7] As noted earlier, these input values aim at averages rather than upper percentiles and median values as well as the mid-age of each group is considered adequate for this purpose.

- the arithmetic means of the French 'Nutri-bébé' survey (boys/girls): 5.7/5.4 kg at 0.5-3 months of age, 7.1/6.5 kg at 4 months of age, 7.8/7.0 kg at 5 months of age and 8.9/8.9 kg at 8-9 months of age [15].
- US data (boys and girls combined) with medians of 5.9 kg (1-<3 months of age) and 9.3 kg (6-12 months of age).

The following table summarizes the input values and results for the alternative approach.

Table S9 Alternative approach for nappies

Age group	Single nappy weight (g)	Use frequency (1/d)	Weight leg bands (g/d)*	Amount migrated (mg/d)**		Body weight (kg)	Dermal exposure (mg/(kg bw x d))		Dermal RCR	
				Mean	Maximum		Mean	Maximum	Mean	Maximum
0-5 mo.	24	7.98	4.79	0.0144	0.0215	5.87	0.00245	0.00367	0.0144	0.0216
6-12 mo.	33	6.66	5.49	0.0165	0.0247	8.79	0.00188	0.00281	0.0110	0.0165

* Calculated: weight of nappy (g) x frequency (1/d) x weight fraction of leg bands. ** Calculated: weight leg bands (g/d) x concentration Hostanox® O 3 (%) x migration rate (%) x 1000 mg/g.

S2.3 Exposure from the use of sealants and other potential consumer products

The detailed exposure estimate results are included in Tables S4-S6. The following table summarizes the resulting RCRs for the three products assessed based on mean and maximum Hostanox® concentrations.

Table S10 Mean and maximum RCRs for the three products assessed

	Mean	Maximum
**Joint sealant (adults)**		
Inhalation RCR	3.38E-10	1.18E-09
Dermal RCR (corrected)	1.85E-02	6.46E-02
Combined RCR	1.85E-02	6.46E-02
**Carpet glue (adults)**		
Inhalation RCR	6.55E-08	2.28E-07
Dermal RCR (corrected)	3.32E-02	1.16E-01
Combined RCR	3.32E-02	1.16E-01
**Bottled glue (toddlers)**		
Inhalation RCR	1.28E-08	4.45E-08
Dermal RCR	3.06E-02	1.06E-01
Combined RCR	3.06E-02	1.06E-01

S2.4 Aggregate exposure and risk from all sources

Contribution of different pathways to aggregate risk

The contribution of the different pathways of exposure to the aggregate RCR is similar across population groups, when adolescents and the very elderly are excluded due to overestimates for exposure via food (see section S0 above) and when the consumer use by adults is removed from the evaluation (aggregate RCR = 0.0611; see main text), since it does not apply to other population groups. Table S11 provides the percentage contribution of each pathway to the aggregate RCR for each population group. On average, oral exposure via food and drinking water and dermal exposure via textiles each account for about 50% of the aggregate RCR. A major deviation is only noted for the

elderly, for whom exposure via textiles accounts for almost 60% of the aggregate RCR due to the comparatively low contribution of exposure via food and drinking water, which in turn results from the low consumption of canned food and soft drinks discussed above (also see Table 3 in the main text). With respect to oral exposure, drinking water contributes a higher fraction than food in all population groups except adults (equal share), again reflecting the consumption data (Table 3 in the main text).

Table S11 Fractions of pathway-specific RCRs of the aggregate RCRs by population group

Population group*	Food**	Drinking water	Textiles
Infants	19%	33%	47%
Toddlers	22%	29%	49%
Other children	21%	33%	47%
Adults	26%	26%	48%
Elderly	17%	24%	58%
Arithmetic mean	21%	29%	50%
Median	21%	29%	48%
Minimum	17% (elderly)	24% (elderly)	47% (infants, other children)
Maximum	26% (adults)	33% (infants, other children)	58% (elderly)

* Adolescents and the very elderly excluded for reasons discussed in the text. ** Canned food and soft drinks combined.

S3 ADDITIONAL EVALUATIONS

The European Union List of Authorized Substances in plastic FCM (Annex I to Regulation 10/2011/EU, as amended by Regulation (EU) 2024/3190 of 31 December 2024) in the version last updated on 13 October 2025 was downloaded from the ECHA website (https://echa.europa.eu/de/plastic-material-food-contact) on 14 October 2025. The list of Annex I substances contains 1 192 entries, of which 928 have a CAS number and/or EC number assigned (Table S12).

Harmonised classifications (Annex VI to the CLP Regulation) were downloaded from the ECHA website (https://echa.europa.eu/information-on-chemicals/annex-vi-to-clp) on 14 October 2025. The version including up to the 21st adaptation to technical progress (ATP 21) was used, since these harmonised classifications apply from 1 September 2025).

Harmonised classifications for substances authorized in plastic FCMs were automatically compiled based on identical CAS number or – when CAS numbers were not available – based on identical EC numbers. This evaluation focused only on classifications that would result in the identification of a substance as 'most harmful chemical' with respect to human health endpoints, as defined in a recent communication from the European Commission [17], i.e. substances classified as

- CMR substances (carcinogenic, mutagenic or reprotoxic, Cat. 1A and 1B),
- endocrine disruptors for human health (Cat. 1),
- respiratory sensitizers (Cat. 1) or
- causing specific target organ toxicity following repeated exposure (STOT-RE Cat. 1).

Annex VI to the CLP Regulation in the version evaluated does yet include any substances classified for endocrine disruption for human health. The following table summarizes the evaluation.

Table S12 Evaluation of authorized substances in plastic FCM

Total number of entries	1192
Number of entries with CAS and/or EC number	928
Number of entries with CAS and/or EC number with harmonised classification	231
Most harmful substances for human health	55
Most harmful substances for human health having a harmonised classification for...	
CMR 1 only	32
of which are classified for Carc. 1 only	11
of which are classified for Repr. 1 only	18
of which are classified for Carc. 1 & Muta. 1	3
STOT RE 1 only	3
Resp. Sens. 1 only	11
CMR 1 & STOT RE 1	8
STOT RE 1 & Resp. Sens. 1	1

The evaluation based on 231 entries with a harmonised classifications shows that 55 (24%) meet the criteria for identification as 'most harmful substances'. This fraction is considered an underestimate for the following reasons:

- Annex I to Regulation 10/2011/EU contains 264 entries without a CAS or EC number that could not be evaluated. Some of these entries, upon more in-depth evaluation, may prove to represent substances with a harmonised classification for relevant endpoints.
- Annex VI to the CLP Regulation contains group entries for which several CAS numbers are provided in the same field (the same applies to EC numbers). Such entries were not accessible by our automated evaluation.

- The definition of a 'most harmful chemical' in the European Commission communication is not based on harmonised classifications but on whether a substance has one or more of the '*hazard properties*' [17] listed above. It remains uncertain what this term means, but since reference is made to hazard classes and categories of the CLP Regulation, it is evident that 'hazard properties' relate to classifications according to the CLP Regulation. However, this may include self-classifications performed by industry under this legislation that we did not evaluate in this analysis, since such an in-depth evaluation was outside the scope of this study.

Additional analyses show that 43 of the 55 Annex I substances meeting the criteria as 'most harmful substances' (78%) are also included in the EU positive list of substances used in materials in contact with drinking water (details not shown).

The fraction of substances meeting the criteria as 'most harmful substances' is likely to increase since more and more substances are classified for relevant properties. If the harmonised classification for Hostanox® O 3 as Repr. 1B recommended by ECHA's RAC is adopted by the European Commission, it would be one a case in point, since the substance is included in both Annex I to Regulation 10/2011/EU as well as in the EU positive list of substances used in materials in contact with drinking water (see main text for details). In addition, substances will be classified for endocrine disruption for human health in the future, also potentially increasing the fraction of substances meeting the criteria as 'most harmful chemicals'.

The evaluations above did not involve substantial data curation. For example, no attempt was made to remove entries with duplicate CAS numbers, which may result in some double counting. Limited additional analyses demonstrate that the fraction of entries meeting the criteria as 'most harmful substances' is slightly lower (21% vs. 24%) when each CAS number is only counted once.

The comparatively high fraction of substances meeting the criteria as 'most harmful substances' must be put into perspective. The fact that these substances are authorized in plastic FCM does not mean that they are not regulation. For example, specific migration limits (SML) are defined for most of these substances and additional restrictions apply in some cases (as defined in Annex I to Regulation 10/2011/EU).

S4 REFERENCES

1. EFSA, European Food Safety Authority. Opinion of the Scientific Panel on food additives, flavourings, processing aids and materials in contact with food (AFC) on a request related to a 13th list of substances for food contact materials. *EFSA J.* **2006**, *4*, 1–25.

2. EFSA CEF Panel, EFSA Panel on Food Contact Materials, Enzymes, Flavourings and Processing Aids. Scientific opinion on recent developments in the risk assessment of chemicals in food and their potential impact on the safety assessment of substances used in food contact materials. *EFSA J.* **2016**, *14*, 4357. https://doi.org/10.2903/j.efsa.2016.4357.

3. FABES. *Migration Study of Hostanox O3;* Report 754/05; FABES-Forschungs-GmbH under contract of Clariant GmbH: München, Germany, 2005.

4. EFSA, European Food Safety Authority. EFSA Comprehensive European Food Consumption Database. Available online: https://www.efsa.europa.eu/en/data-report/food-consumption (accessed on 8-16 January 2024).

5. EFSA, European Food Safety Authority. Use of the EFSA Comprehensive European Food Consumption Database in Exposure Assessment. *EFSA J.* **2011**, *9*, 2097. https://doi.org/10.2903/j.efsa.2011.2097.

6. EFSA, European Food Safety Authority. Guidance on selected default values to be used by the EFSA Scientific Committee, Scientific Panels and Units in the absence of actual measured data. *EFSA J.* **2012**, *10*, 2579. https://doi.org/10.2903/j.efsa.2012.2579.

7. Oltmanns, J.; Macherey, M.; Schwarz, M.; Manžuch, Z.; Hayleck, M.; Heine, K. *Mapping of Data Requirements and Assessment Methodologies Linked to the Regulatory Frameworks and Remits of the Relevant EU Agencies (ECHA, EFSA and EMA) and EC Scientific Committees (SCCS and SCHEER): Final Report*; EFSA Supporting Publication: Parma, Italy, 2023; p. 264.

8. EFSA/EMA, European Food Safety Authority/European Medicines Agency. *Report on Development of a Harmonised Approach to Human Dietary Exposure Assessment for Residues from Veterinary Medicinal Products, Feed Additives and Pesticides in Food of Animal Origin*; European Medicines Agency: Amsterdam, The Netherlands; European Food Safety Authority: Parma, Italy, 2022.

9. BfR, Bundesinstitut für Risikobewertung. Einführung in die Problematik der Bekleidungstextilien. Aktualisierte Stellung-nahme Nr. 041/2012 des BfR vom 6. Juli 2012. 2012. Available online: http://www.bfr.bund.de/cm /343/einfuehrung-in-die-problematik-der-bekleidungstextilien.pdf (accessed on 6 October 2025).

10. US EPA, Environmental Protection Agency. *Exposure Factors Handbook: 2011 Edition*; EPA/600/R-090/052F; U.S. Environmental Protection Agency, National Center for Environmental Assessment, Office of Research and Development: Washington, DC, 2011.

11. Phillips, L.J.; Fares, R.J.; Schweer, L.G. Distributions of total skin surface area to body weight ratios for use in dermal exposure assessments. *J. Expo. Anal. Environ. Epidemiol.* **1993**, *3*, 331–338.

12. Cieszynski, A.; Jung, C.; Schendel, T.; ter Burg, W. *Do-It-Yourself Products Fact Sheet. Updated version 2022*; RIVM Report 2022-0208; RIVM, Rijksinstituut voor Volksgezondheid en Milieu: Bilthoven, The Netherlands, 2022.

13. te Biesebeek, J.D.; Nijkamp, M.M.; Bokkers, B.G.H.; Wijnhoven, S.W.P. *General Fact Sheet. General Default Parameters for Estimating Consumer Exposure—Updated Version 2014*; RIVM Report 090013003/2014; National Institute for Public Health and the Environment: Bilthoven, The Netherlands, 2014. Available online: http://www.rivm.nl/dsresource?objectid=fd9e2199-e7ad-4641-8b97-aff66699c0c4&type=org&disposition=inline (accessed on 7 October 2025).

14. ANSES, Agence nationale de sécurité sanitaire de l'alimentation, de l'environnement et du travail. *Sécurité des couches pour bébé Avis révisé de l'Anses Rapport d'expertise collective. Avis de l'Anses Saisine n° 2017-SA-0019*; France: 2019.

15. Chouraqui, J.P.; Tavoularis, G.; Emery, Y.; Francou, A.; Hébel, P.; Bocquet, M.; Hankard, R.; Turck, D. The French national survey on food consumption of children under 3 years of age—Nutri-Bébé 2013: Design, methodology, population sampling and feeding practices. *Public Health Nutr.* **2018**, *21*, 502–514. https://doi.org/10.1017/s1368980017002518.

16. RKI, Robert Koch-Institut. *Referenzperzentile für Anthropometrische Maßzahlen und Blutdruck aus der Studie zur Gesundheit von Kindern und Jugendlichen in Deutschland (KiGGS) 2. Erweiterte Auflage*; Beiträge zur Gesundheitsberichterstattung des Bundes; Robert Koch-Institut: Berlin, Germany, 2013.

17. EC, European Commission. *Communication from the Commission—Guiding Criteria and Principles for the Essential Use Concept in EU Legislation Dealing with Chemicals*; C/2024/2894; European Commission: Brussels, Belgium, 2024. Available online: https://eur-lex.europa.eu/legal-content/EN/TXT/?uri=OJ%3AC_202402894 (accessed on 18 November 2025).
